# The PINK1 kinase-driven ubiquitin ligase Parkin promotes mitochondrial protein import through the presequence pathway in living cells

**DOI:** 10.1038/s41598-019-47352-9

**Published:** 2019-08-14

**Authors:** M. Jacoupy, E. Hamon-Keromen, A. Ordureau, Z. Erpapazoglou, F. Coge, J.-C. Corvol, O. Nosjean, C. Mannoury la Cour, M. J. Millan, J. A. Boutin, J. W. Harper, A. Brice, D. Guedin, C. A. Gautier, O. Corti

**Affiliations:** 1 Inserm, U1127, F-75013 Paris, France; 2 CNRS, UMR 7225, F-75013 Paris, France; 3 Sorbonne Universités, UPMC Univ Paris 06, UMR S 1127, F-75013 Paris, France; 4 Institut du Cerveau et de la Moelle épinière, ICM, F-75013 Paris, France; 5 Department of Cell Biology, Harvard Medical School, Boston, MA 02115 USA; 6 Laboratoire de Chémogénétique Servier, F-75013 Paris, France; 7 Institut de Recherches Servier, Croissy-sur-Seine, France; 8 Assistance-Publique Hôpitaux de Paris, Inserm, CIC-1422, Department of Neurology, Hôpital Pitié-Salpêtrière, F-75013 Paris, France

**Keywords:** Mechanisms of disease, Mitochondria, Parkinson's disease

## Abstract

Most of over a thousand mitochondrial proteins are encoded by nuclear genes and must be imported from the cytosol. Little is known about the cytosolic events regulating mitochondrial protein import, partly due to the lack of appropriate tools for its assessment in living cells. We engineered an inducible biosensor for monitoring the main presequence-mediated import pathway with a quantitative, luminescence-based readout. This tool was used to explore the regulation of mitochondrial import by the PINK1 kinase-driven Parkin ubiquitin ligase, which is dysfunctional in autosomal recessive Parkinson’s disease. We show that mitochondrial import was stimulated by Parkin, but not by disease-causing Parkin variants. This effect was dependent on Parkin activation by PINK1 and accompanied by an increase in the abundance of K11 ubiquitin chains on mitochondria and by ubiquitylation of subunits of the translocase of outer mitochondrial membrane. Mitochondrial import efficiency was abnormally low in cells from patients with *PINK1-* and *PARK2*-linked Parkinson’s disease and was restored by phosphomimetic ubiquitin in cells with residual Parkin activity. Altogether, these findings uncover a role of ubiquitylation in mitochondrial import regulation and suggest that loss of this regulatory loop may underlie the pathophysiology of Parkinson’s disease, providing novel opportunities for therapeutic intervention.

## Introduction

Mitochondria fulfill crucial functions in energy production and metabolism, constitute platforms for the development of protective homeostatic responses to cellular stress, and play a key role in the control of apoptotic cell death. Mitochondria contain over a thousand proteins, almost 99% of which are encoded by nuclear genes. They are synthesized as precursors on cytosolic ribosomes, then transferred into the organelle by the translocase of the outer mitochondrial membrane (TOM), for sorting to their final destination in one of the four submitochondrial compartments by specific molecular machineries^1^. The disruption of mitochondrial import is thought to underlie various neurodegenerative diseases, including Alzheimer’s disease, amyotrophic lateral sclerosis, Huntington’s disease and Parkinson’s disease (PD)^2–8^. The species of α-synuclein accumulating in the brains of most PD patients have been shown to bind with high affinity to the TOM20 subunit of the TOM complex, thereby impairing protein import and mitochondrial function^8^. The mitochondrial serine/threonine kinase PINK1 and the cytosolic ubiquitin (Ub)-protein ligase Parkin, which are dysfunctional in autosomal recessive PD, function together as a molecular quality control system, coupling the loss of mitochondrial import efficiency via the TOM machinery to the degradation of dysfunctional mitochondria (mitophagy) under conditions of severe mitochondrial stress^9^. This process involves the stabilization of PINK1 in close proximity to the TOM complex, the recruitment, and activation of Parkin through the Ser65 phosphorylation of Ub and the Ub-like domain of Parkin, and the Parkin-mediated ubiquitylation of a series of outer mitochondrial membrane (OMM) proteins^9–13^. PINK1 and Parkin also regulate several aspects of mitochondrial biogenesis: they promote the degradation of PARIS/ZNF746, a transcriptional repressor of the master regulator of mitochondrial biogenesis, proliferator-activated receptor gamma coactivator-1-alpha (PGC-1-α)^14,15^; and they control the localized translation on the OMM of mRNAs encoding specific nuclear genome-encoded respiratory chain components (RCCs)^16^. Moreover, PINK1 and Parkin interact with the mitochondrial matrix enzyme 17-β**-**hydroxysteroid dehydrogenase 10 (HSD17B10) at the TOM complex, and we hypothesized that this interaction facilitates the translocation of HSD17B10 into the organelle^17^. However, whether PINK1 and Parkin can modulate mitochondrial import has not been reported so far.

**Figure 1:**
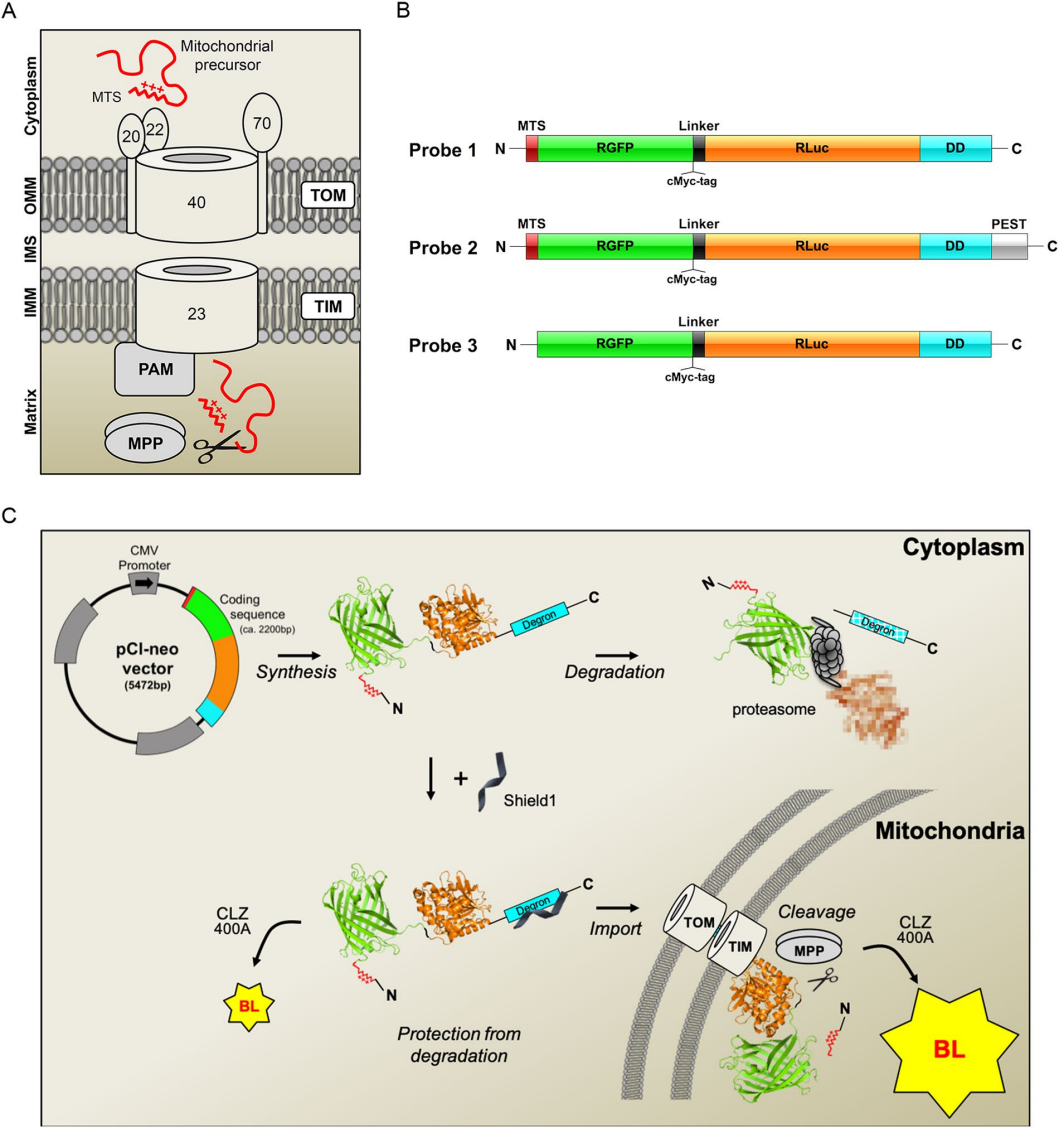
Genetically encoded reporters for the assessment of protein import through the TOM complex. (**A**) Molecular components of the presequence import pathway: translocase of the outer mitochondrial membrane (TOM) with its receptor subunits TOM20, TOM22 and the channel subunit TOM40; translocase of inner mitochondrial membrane (TIM23); presequence translocase-associated motor (PAM); mitochondrial processing peptidase (MPP). OMM, outer mitochondrial membrane; IMM inner mitochondrial membrane; IMS, intermembrane space. The TOM70 receptor recognizes proteins with internal mitochondrial targeting information. (**B**) Probes designed to monitor import through the presequence pathway. MTS, mitochondrial targeting signal; RGFP, green fluorescent protein from *Renilla reniformis*; RLuc, luciferase from *Renilla reniformis;* DD, destabilizing domain of FK506 binding protein (FKBP); PEST, sequence rich in proline (P), glutamic acid (E), serine (S), and threonine (T) associated with short-lived proteins; the D433A and D434A PEST mutations accelerate degradation. (**C**) Bioluminescence (BL)-based assay for mitochondrial import through the presequence pathway. In the absence of the small molecule Shield1, Probes 1 and 2 are rapidly degraded by the proteasome. Shield1 stabilizes the probes, which are then imported into the mitochondrial matrix through the TOM and TIM complexes. Light emission in the cytoplasm is weak, due to the presence of the MTS, blocking the N-terminus of RGFP. Cleavage of the MTS by MPP allows interaction between the RGFP and RLuc modules, leading to a characteristic light emission due to resonance energy transfer and quantum yield enhancement in the presence of the Rluc substrate coelenterazine 400A (CLZ400A). The RGFP and Rluc protein modules (PDB ID: 2RH7^78^ and PDB ID: 2PSD^78^) were obtained from the Protein Data Bank^79^ at the following URL: http://www.rcsb.org.

Two-thirds of all mitochondrial proteins are targeted to the mitochondria by a positively charged N-terminal mitochondrial targeting signal (MTS), via the presequence pathway, in which they are recognized by the TOM20 and TOM22 receptors, transferred to the TOM40 channel and directed to the presequence translocase of the inner mitochondrial membrane (TIM). The proteins are pulled into the matrix through the TIM23 channel, in a process dependent on mitochondrial transmembrane potential (Δψ_mit_), the presequence translocase-associated motor (PAM) and the ATP-dependent mitochondrial heat-shock protein 70. They are then generally processed by the matrix-resident mitochondrial processing peptidase MPP, which removes the MTS (Fig. 1A). We developed a novel tool for assessing the roles of PINK1 and Parkin in the regulation of this import pathway. We used the properties of fusions between the *Renilla reniformis* green fluorescent and luciferase proteins (RGFP, RLuc)^18^ to engineer a molecular biosensor targeted to mitochondria by a classical MTS and generating a robust bioluminescent signal only after import into the organelle and removal of the MTS. Using this molecular biosensor in a bioluminescence-based cellular assay, we found that PINK1 and Parkin jointly facilitated mitochondrial protein import through the TOM/TIM complexes. We also found that mitochondrial protein import efficiency was abnormally low in cells derived from PD patients with *PARK2* or *PINK1* mutations, suggesting that the loss of this regulatory function of PINK1 and Parkin contributes to the pathophysiology of PD.

## Results

### Design of a biosensor for the assessment of the mitochondrial presequence import pathway

In the marine anthozoan Renilla bioluminescence is generated through a high-efficiency resonance energy transfer process resulting from specific protein-protein interaction between RLuc and RGFP, converting most of the blue light emitted by the excited RLuc substrate coelenterazine (CLZ) into green light^19,20^. RGFP binding to RLuc is associated with an enhancement in photon yield, probably linked to reversal of quenching of excited CLZ states in the presence of RLuc only, and/or to changes to RLuc conformation that optimize catalytic activity^21^. The gain in light output in the presence of RGFP is maximized *in vitro* when the RLuc reaction is triggered by low-quantum yield CLZ analogs, such as CLZ400A^21^. This system is also functional in mammalian cells when RGFP and RLuc are coexpressed as a single fusion protein, with the RGFP module placed at the N-terminus^18^. However, its efficiency is greatly reduced by short N-terminal extensions to the RGFP module^18^. Based on these principles, we designed two inducible modular biosensors (Probes 1 and 2) predicted to yield a strong bioluminescent signal in the presence of CLZ400A only after import and the subsequent proteolytic removal of the MTS in the mitochondrial matrix by MPP (Fig. 1B,C). Each of these probes consisted of (1) an N-terminal cleavable MTS from the human dihydrolipoamide dehydrogenase (DLD) directly fused to (2) RGFP, followed by (3) a linker containing a cMyc tag, (4) Rluc, and (5) a C-terminal conditional FKBP-based destabilizing domain (DD) targeting the probe for proteasomal degradation under basal conditions, and stabilizing it in the presence of the small molecule Shield1^22^. Probe 2 also contained a PEST motif from mouse ornithine decarboxylase to accelerate its degradation, as a means of optimizing the signal-to-noise ratio and limiting potential toxicity^23,24^. As a control, we generated a cytosolic probe based on the backbone of Probe 1 but lacking the MTS (Probe 3).

### The biosensor allows monitoring of changes to mitochondrial import efficiency in living cells

**Figure 2:**
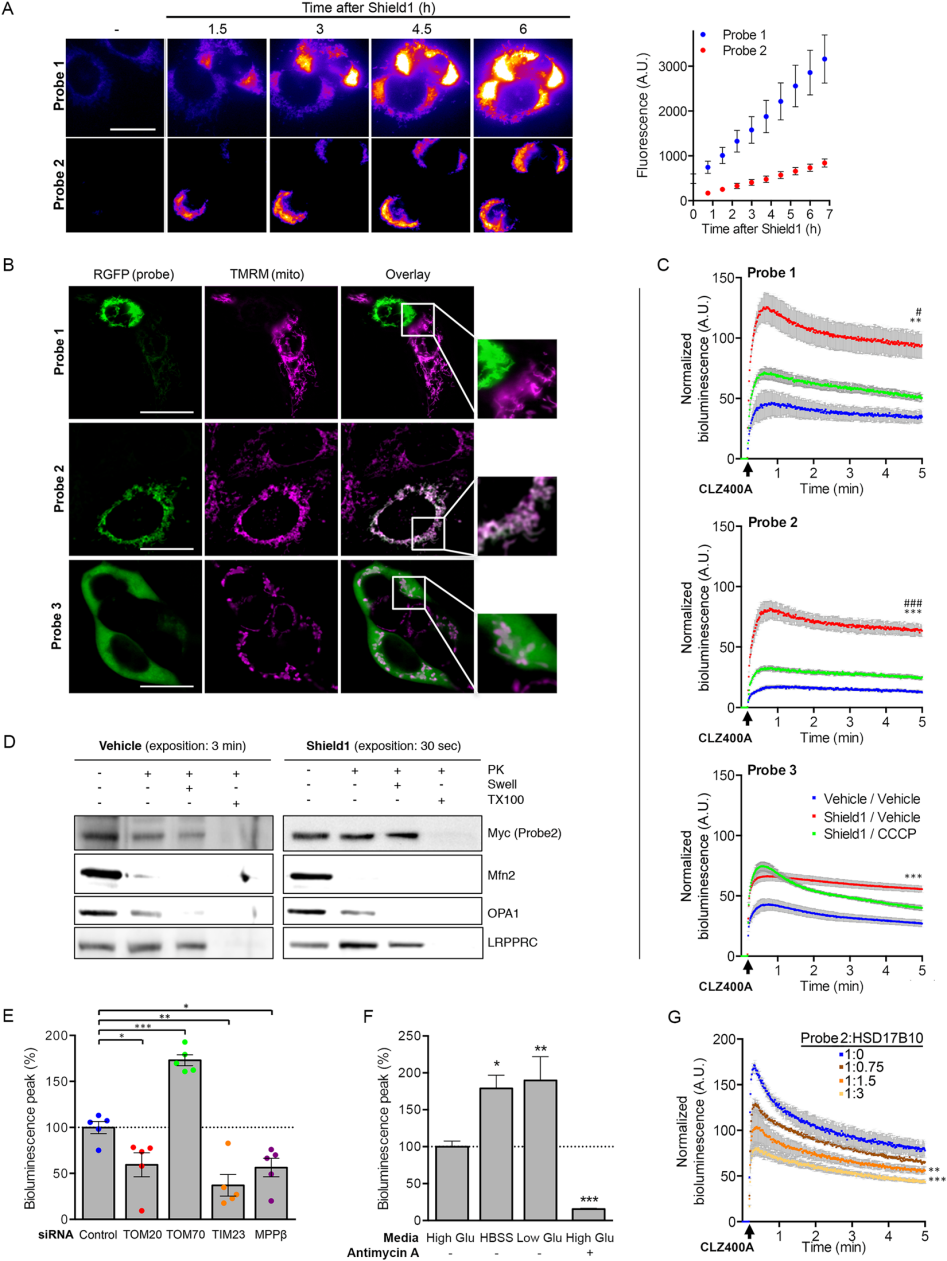
Validation of the probes for the assessment of the presequence import pathway in living cells. (**A**) Representative fluorescence images and the corresponding quantitative analysis (graph), illustrating the expression of Probes 1 and 2 (RGFP signal) at different time points after the addition of Shield1 to the culture medium of HEK293T cells. *n* = 15 (**B**) Representative fluorescence images and higher magnifications (framed regions in the overlay) showing the colocalization of each probe (RGFP signal) with TMRM in cells treated with Shield1 (24 h). Note the loss of TMRM staining in a cell with high levels of Probe 1, and the expected cytosolic localization of Probe 3. (**C**) Quantitative analysis of the bioluminescent signals emitted by Probes 1-3 in HEK293T cells with and without Shield1 and/or CCCP treatment (24 h), following the addition of CLZ400A. *n* = 6 wells from one of three independent experiments. (**D**) Western blot analysis of mitochondrial subfractions obtained by subjecting mitochondrion-enriched fractions to proteinase K (PK) treatment, combined or not with osmotic swelling (Swell) or solubilization with Triton X‐100 (TX100). The subfractions were probed for Mfn2 (OMM), OPA1 (IMS/IMM), LRPPRC (matrix). Note the presence of Probe2 in the mitochondrial matrix. (**E**–**G**) Changes to bioluminescent signal emitted by Probe 2 in HEK293T cells following (**E**) siRNA-mediated silencing of key components of the presequence pathway (*n* = 5 independent experiments), (**F**) switch to nutrient-free (HBSS) or low glucose medium (1 g/l), or treatment with antimycin A (*n* = 3 independent experiments), or (**G**) cotransfection with various amounts of plasmid encoding HSD17B10 (V5 epitope-tagged; *n* = 5 wells from one of at least three independent experiments). Results are expressed as means ± SEM. *p < 0.05, **p < 0.01, ***p < 0.001 versus vehicle/vehicle (**C;** two-way repeated-measures ANOVA with Tukey’s posthoc test), Control (**E**; one-way ANOVA with Dunnett’s posthoc test), High Glu (**E**; one-way ANOVA with Dunnett’s posthoc test) or 1:0 ratio of Probe 2 to HSD17B10-V5 (**F**, two-way repeated-measures ANOVA with Dunnett’s posthoc test). ^#^*p* < 0.05, ^###^*p* < 0.001 versus Shield1/CCCP. Scale bar: 10 µm.

We validated the probes in transiently transfected HEK293T cells. Monitoring of the fluorescent signal after the addition of Shield1 to the culture medium revealed a time-dependent increase in fluorescence for Probes 1 and 2, with Probe 1 having both a stronger signal and higher background in the absence of induction (Fig. 2A). The colocalization of the probes with the mitochondrial fluorescent dye tetramethylrhodamine methyl ester (TMRM) confirmed that they were targeted to the mitochondria (Fig. 2B). In cells expressing high levels of Probe 1, the TMRM signal was very weak, indicating a decrease in Δψ_mit_, possibly due to the reported toxicity of RGFP at high concentrations^25^. We then set up an assay for quantitative evaluation of the bioluminescent probe signals and their dependence on mitochondrial protein import in living cells. The addition of CLZ400A to the cell culture medium triggered the emission of the expected bioluminescent signal in the presence of Shield1 (Fig. 2C). Treatment with the protonophore carbonyl cyanide 3-chlorophenylhydrazone (CCCP), which dissipates Δψ_mit_ and, thus, the driving force for import, greatly decreased the intensity of the bioluminescent signals of Probes 1 and 2. By contrast, the signal of Probe 3 was not affected by CCCP, and the probe was found in the cytosol (Fig. 2B,C).

We used Probe 2 in most of the subsequent experiments because it provided the best signal-to-noise ratio. Subfractionation of mitochondria isolated from transiently transfected HEK293T cells treated or not with Shield1, by combining treatment with trypsin (Supplementary Fig. 1A) or proteinase K with osmotic swelling or membrane solubilization (Fig. 2D), provided biochemical evidence for the expected localization of the probe in the mitochondrial matrix. Complementary *in vitro* import assays confirmed translocation of the probe into the organelle (Supplementary Fig. 1B). In addition, as expected, the probe was stabilized by Shield1 and proteasome inhibition (Supplementary Fig. 1A and C). We further validated its specificity by assessing the impact of siRNA-mediated silencing of components of the presequence mitochondrial protein import pathway: the TOM20 receptor, the channel subunit of the TIM23 complex, and the β subunit of the protease MPP (Fig. 2E and Supplementary Fig. 1D). In all cases, the intensity of the signal was decreased by 40 to 60%, consistent with lower levels of import. Under these experimental conditions, the abundances of proteins representative of the various submitochondrial compartments remained similar, excluding more global effects on mitochondrial turnover (Supplementary Fig. 1E). Intriguingly, downregulation of the peripheral TOM70 receptor, which recognizes proteins with internal mitochondrial targeting information, increased the intensity of the probe signal by almost 70%. This suggests that there is competition between the TOM20- and TOM70-dependent pathways, through the shared TOM40 channel, and that TOM subcomplexes with a lower molar ratio of TOM70 to TOM40^26^ may translocate MTS-carrying precursors more efficiently.

We then evaluated the effect of conditions known to affect mitochondrial function on the import of the probe. In mammalian cells, nutrient starvation is associated with autophagy induction, accompanied by mitochondrial elongation, inhibition of mitophagy and optimization of ATP production^27,28^. Moreover, in HEK293 cells, amino acid starvation enhances mitochondrial protein synthesis and mitochondrial respiration^29^. Incubation of cells expressing Probe 2 in nutrient-free medium (HBSS) resulted in a nearly 100%, enhancement of bioluminescence emission, consistent with increased protein import (Fig. 2F). A similar effect was observed in cells grown in the presence of low concentrations of glucose. In contrast, the mitochondrial complex III inhibitor, antimycin A, attenuated the signal by more than 80%. Finally, we evaluated the effect of increasing amounts of HSD17B10, which we thought might interfere with the mitochondrial import of Probe 2, as it is also imported via the presequence pathway^30^. HSD17B10 overproduction led to a dose-dependent decrease in the bioluminescent signal of Probe 2, indicating competition for import through the TOM20-dependent pathway (Fig. 2G and Supplementary Fig. 1F).

### Use of the biosensor uncovers regulation of mitochondrial protein import by the PINK1/Parkin system

**Figure 3:**
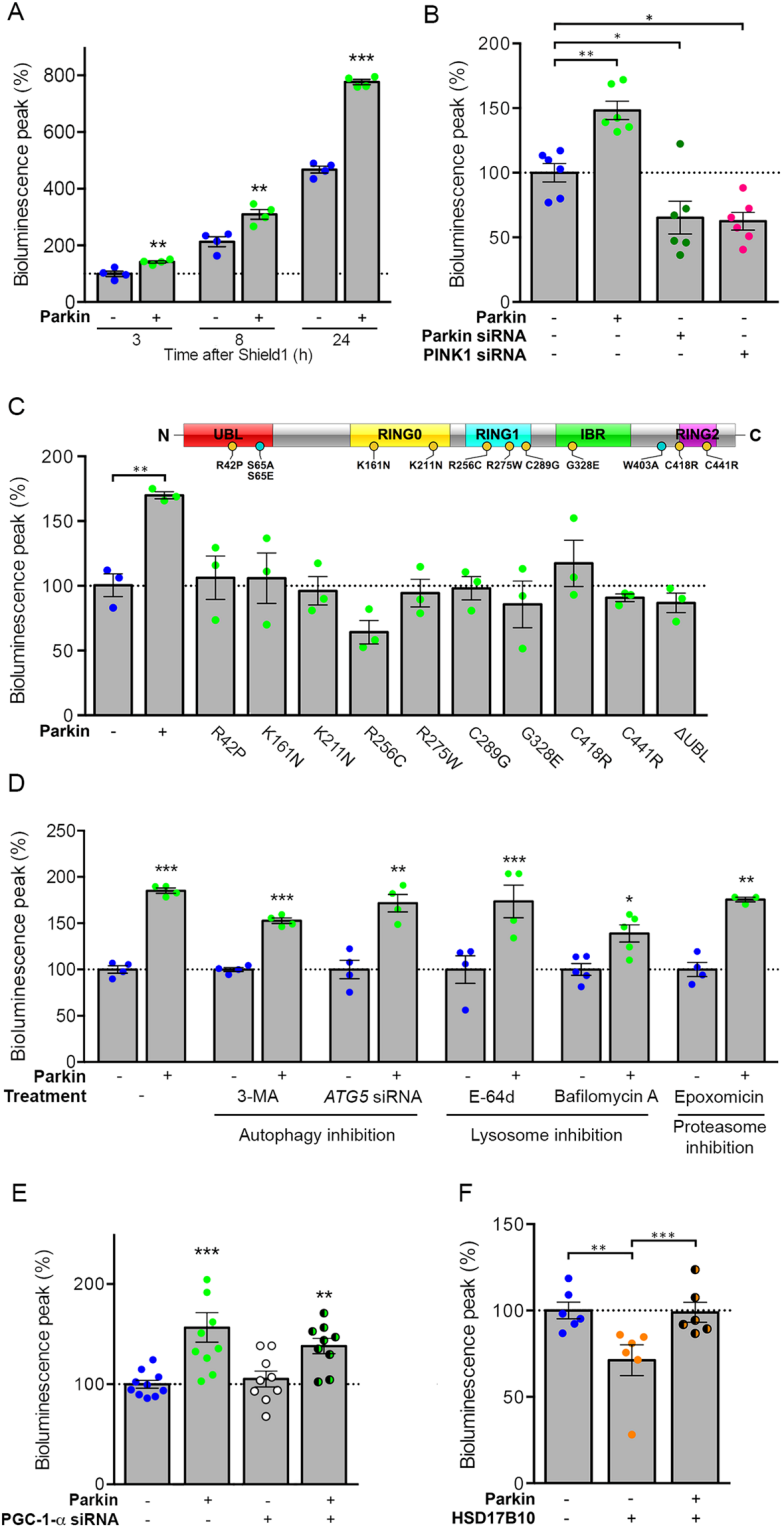
Parkin modulates the mitochondrial presequence import pathway independently of its role in mitophagy. Quantitative analyses of the relative bioluminescent signals for Probe 2 in HEK293T cells (**A**) overproducing Parkin after 3, 8 or 24 h of treatment with Shield1, (**B**) silenced for Parkin or PINK1 or (**C**) overproducing pathogenic Parkin variants, showing the enhancement of import by normal Parkin but not by PD-linked or artificial (ΔUbl) variants. **(D**) Inhibition of autophagy (3-Methyladenine, *ATG5* silencing), lysosomal (E-64d, Bafilomycin A1) or proteasomal (Epoxomicin) functions, or (**E**) siRNA-mediated silencing of *PGC-1-α* siRNA do not affect facilitation of mitochondrial import by Parkin. (**F**) Overproduction of Parkin is sufficient for rescuing the mitochondrial import decrease of the probe following HSD17B10 overproduction. Results are expressed as means ± SEM. *n* = 4 (**A**) to 6 (**B**) independent experiments. Data were analyzed by student t-test (**A**,**D**) one-way (**C**) or two-way ANOVA (**B**,**E**,**F**) with Dunnett’s or Holm-Sidak’s posthoc tests. *p < 0.05, **p < 0.01, ***p < 0.001. The schematic diagram illustrates Parkin with its main domains and the location of the PD-linked (yellow circles) or artificial (blue circles) variants examined.

We used Probe 2 to investigate the possibility that Parkin and PINK1 coregulate the presequence import pathway. Parkin overproduction in HEK293T cells increased the bioluminescence emission of Probe 2 by almost 50%, as early as 3 h after induction by Shield1 and until at least 24 h (Fig. 3A and Supplementary Fig. 2A). In contrast, the downregulation of Parkin or PINK1 had the opposite effect, suggesting that these proteins work together to facilitate mitochondrial protein import (Fig. 3B and Supplementary Fig. 2B). This effect was observed whether the bioluminescence was normalized against the global fluorescence of the probe or the intensity of the MitoTracker signal; it was not, therefore, related to general changes in mitochondrial abundance (Supplementary Fig. 2C). Probe 2 import was not modified by a UBL domain-deficient Parkin variant, or by any of the nine PD-related variants that we previously found to be unable to associate with the TOM complex in Förster resonance energy transfer (FRET) experiments in cells treated with CCCP^31^ (Fig. 3C and Supplementary Fig. 2D). Most of these variants (K161N, K211N, R275W, C418R, C441R, ∆UBL) have little or no ubiquitylation capacity, based on *in vitro* assays for autoubiquitylation^32,33^, substrate ubiquitylation^34^ or ubiquitin chain assembly^34,35^, and/or show impaired mitochondrial recruitment^35^. Import stimulation by Parkin was independent of its role in mitophagy, as it was preserved in the presence of the autophagy inhibitor 3-methyladenine, following silencing of the autophagy protein 5 (ATG5), in the presence of the lysosome activity inhibitors, Bafilomycin A or E-64d, or upon blockade of the proteasome (Epoxomicin), which cooperates with autophagy in the degradation of dysfunctional mitochondria^31,36–38^ (Fig. 3D and Supplementary Fig. 2E). Moreover, silencing of PGC-1-α did not modify the signal in cells overproducing Parkin or with endogenous Parkin levels, indicating that changes in bioluminescence levels are not accounted for by changes in mitochondrial biogenesis in our experimental conditions (Fig. 3E and Supplementary Fig. 2F). Finally, Parkin overproduction reversed the mitochondrial import defects associated with the production of large amounts of HSD17B10 (Fig. 3F and Supplementary Fig. 2G).

**Figure 4:**
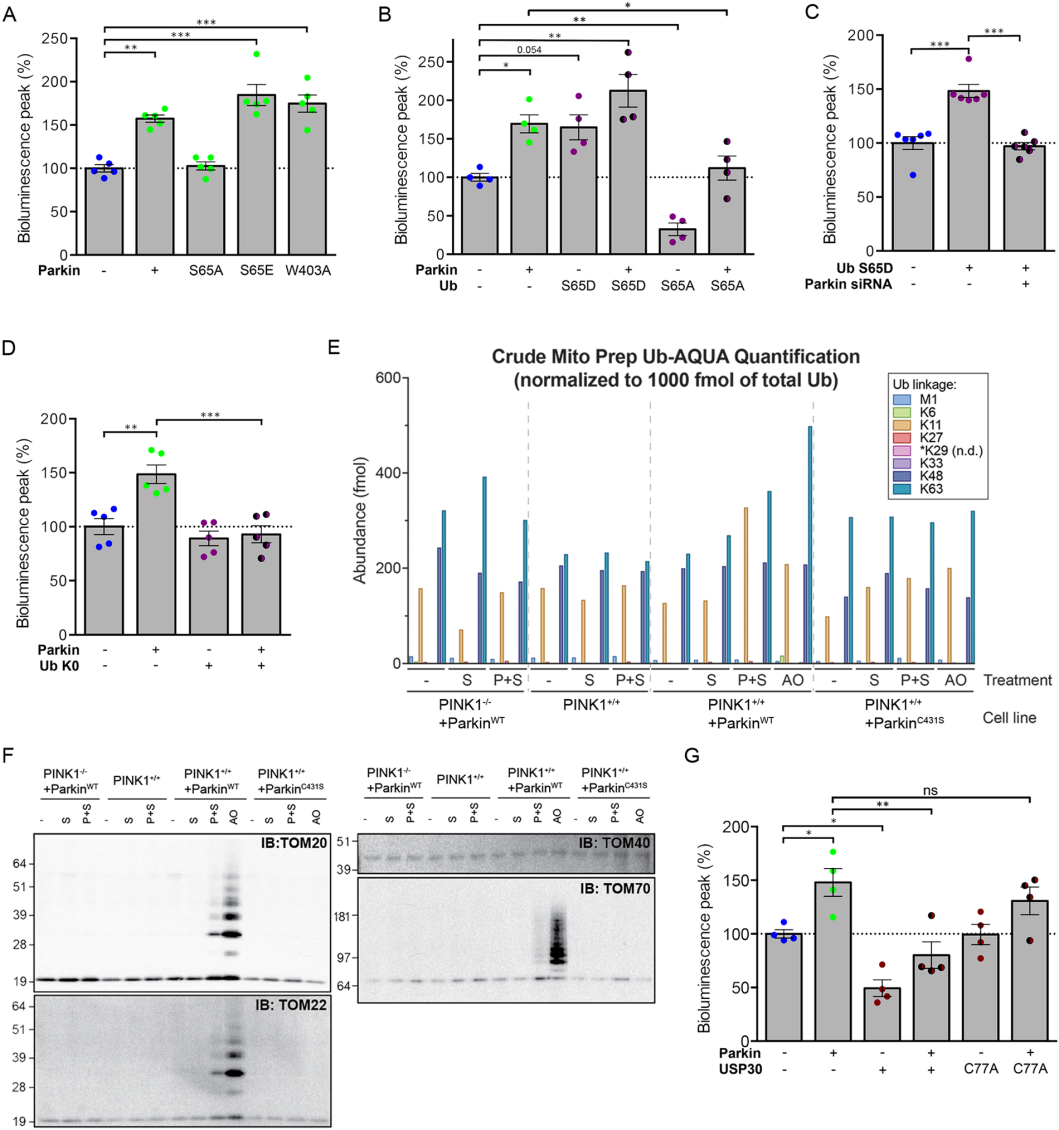
Parkin modulates mitochondrial import through its E3 ubiquitin-protein ligase activity induced by PINK1. Effect of relief of Parkin autoinhibition by (**A**) the activating W403A substitution, or by mimicking the PINK1-mediated phosphorylation of Parkin UBL (S65E) or (**B**) Ub (S65D), showing exacerbation of the Parkin-dependent enhancement of the bioluminescent signal of Probe 2 in HEK293T cells. By contrast, non-phosphorylatable S65A Parkin or Ub variants abolish the effect of Parkin. (**C**) In these cells, Ub S65D mimics the effect of Parkin overproduction in a manner dependent on endogenous Parkin (Parkin siRNA). (**D**) Effect of the lysine-less Ub K0 variant on the intensity of the bioluminescent signal of Probe 2 in HEK293T, indicating that the effect of Parkin is mediated by polyubiquitylation. (**E**) Quantification by UB-AQUA proteomics of individual Ub chain linkage types associated with mitochondria in HeLa Flp-In T-REx Parkin^WT^ or Parkin^C431S^ cells depleted or not of PINK1 and expressing Probe 2 in the presence of Shield1 (P + S), or treated with S or AO. (**F**) Immunoblot analysis of ubiquitylated proteins pulled down with TUBEs from mitochondrion-enriched fractions analyzed in (**E**) shows ubiquitylation of the TOM subunits TOM20, TOM22 and TOM70 in cells expressing Probe 2 (P + S). (**G**) Analysis of the impact of the mitochondrial ubiquitin-specific protease USP30 or its inactive C77A variant on the signal of Probe in the presence or absence of overexpressed Parkin. *n* = 4 (**A**,**B**,**G**), 5 (**D**) or 6 (**C**) independent experiments. Results are expressed as means ± SEM. Data were analyzed by one-way (**A**) or two-way ANOVA (**B**,**C**,**G**) with Dunnett’s or Holm-Sidak’s posthoc tests. *p < 0.05, **p < 0.01, ***p < 0.001. ns: non-significant.

We then evaluated the role of the PINK1-dependent activation of the E3 ligase Parkin in import stimulation. The PINK1-dependent phosphorylation of the Ser65 residues of Ub and Parkin plays a crucial role in activating and maintaining the E3 Ub ligase activity of Parkin, through a complex allosteric mechanism triggering conformational changes in an otherwise autoinhibited protein^11–13,35,39–46^. The Parkin W403A mutation also weakens autoinhibition, promoting its E3 ligase activity^39^. Co-production of the Parkin S65A variant, which cannot be phosphorylated, abolished the Parkin-dependent enhancement of the bioluminescent signal emitted by Probe 2, whereas phosphomimetic Parkin S65E or Parkin W403A had a stronger signal-enhancing effect than native Parkin (almost +25% and +15%, respectively; Fig. 4A and Supplementary Fig. 3A). Moreover, production of the phosphomimetic Ub variant S65D enhanced the effect of overproduced Parkin (+40%) and tended to mimic Parkin overproduction in cells with endogenous Parkin levels (Fig. 4B and Supplementary Fig. 3B). Accordingly, the effect of Ub S65D was reversed by Parkin silencing, demonstrating a dependence of its action on the E3 activity of endogenous Parkin (Fig. 4C). By contrast, the non-phosphorylatable Ub S65A decreased the intensity of the Probe 2 signal (−70%), consistent with a dominant negative effect of this variant^13^, and almost abolished the effect of Parkin (only +10% versus +60% without Ub S65A) (Fig. 4B and Supplementary Fig. 3C). Similarly, the overexpression of a lysine-less Ub variant that inhibits ubiquitin-chain extension^47^ prevented the stimulation of import by Parkin (Fig. 4D and Supplementary Fig. 3D).

### Regulation of mitochondrial protein import is associated with ubiquitylation of the TOM complex

To complement our observations, we used absolute quantification (AQUA)-based proteomics to provide direct evidence for Parkin activation in cells expressing Probe 2^48,49^. We previously applied this approach to quantify the relative abundance of distinct Ub chain linkage types (UB-AQUA) on depolarized mitochondria isolated from a HeLa cell line (HeLa Flp-In T-REx) genetically modified to express Parkin in a doxycycline-inducible manner^35^. Here, we transiently expressed Probe 2 in HeLa Flp-In T-REx cells expressing Parkin or the C431S variant lacking the catalytic Cys residue **(**Supplementary Fig. 4A and B) and confirmed mitochondrial import facilitation by Parkin. As a control, the probe was also expressed in these same cell lines homozygously knocked out for PINK1^35^. We compared the profile of Ub chain linkage types observed on mitochondrion-enriched fractions isolated from cells expressing the probe or treated with the mitochondrial complex III and V inhibitors, antimycin A and oligomycin (AO), to depolarize mitochondria (Fig. 4E). Under our experimental conditions, AO treatment caused a ~2-fold increase in the abundance of K63 Ub chains in the presence of normal Parkin, but not in the presence of the Parkin C431S or in absence of PINK1. As expected, Ub chains phosphorylated at S65 were also readily detected in this condition. Following expression of Probe 2, we observed a detectable increase in the abundance of non-canonical K11 Ub chains only in the presence of normal Parkin and PINK1 (Supplementary Fig. 4C), indicating that these chains are formed as a consequence of the PINK1-dependent activation of Parkin. Ub chains phosphorylated at S65 were not detected in this case, consistent with a lower level of Parkin activation compared to that triggered by mitochondrial depolarization.

Subunits of the TOM machinery are among the earliest targets for PINK1-driven Parkin-dependent ubiquitylation in severely damaged mitochondria^17,31,37,38,50,51^. To determine whether stimulation of mitochondrial import by Parkin in cells expressing Probe 2 is accompanied by ubiquitylation of the TOM complex, we used Halo–tandem-repeated Ub-binding entities (TUBEs)^52^ to pull down ubiquitylated proteins from the mitochondrion-enriched fractions isolated from the different HeLa Flp-In T-REx cell lines, in the conditions described above. Western blot against Ub confirmed enrichment of Ub-chains in cells expressing normal Parkin and treated with AO, and albeit at significantly lower levels, in cells coexpressing Parkin and Probe 2 (Fig. 4F and Supplementary Fig. 4D). There was no increase in Ub signal associated with mitochondria from cells expressing Parkin C431S or lacking PINK1. Moreover, the TOM20, TOM22 and TOM70 subunits of the TOM complex were found to be ubiquitylated both in response to AO and to a lower extent in cells expressing Probe 2. In contrast, Mitofusin 2 and CISD1, known to be ubiquitylated in a Parkin-dependent manner following mitochondrial depolarization, were not detected among the ubiquitylated proteins captured from cells expressing Probe 2 but were readily detected in the Ub-enriched fractions from cells treated with AO.

The OMM ubiquitylase USP30 has been shown to antagonize the Parkin-dependent ubiquitylation of proteins, including TOM subunits, on dysfunctional mitochondria and to block mitophagy^51^. We, therefore, determined whether USP30 counteracts the facilitation of protein import by Parkin. We found that USP30 overproduction greatly reduced the bioluminescence of Probe 2, in both the presence (−70% versus + Parkin) and absence (50% versus control conditions) of exogenous Parkin, whereas the catalytically inactive USP30 C77A mutant had no effect (Fig. 4G, Supplementary Fig. 4E and F).

### The biosensor reveals a reduction in mitochondrial protein import in PINK1- and PARK2-linked PD patients

**Figure 5:**
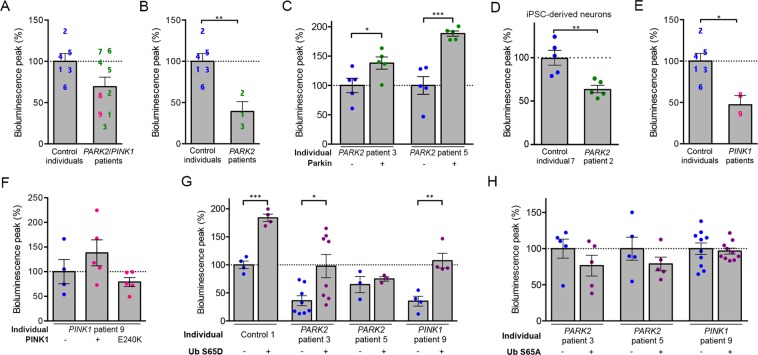
Defects in the mitochondrial presequence import pathway in fibroblasts from PD patients with *PARK2* and *PINK1* mutations are rescued by phosphomimetic ubiquitin. (**A**–**F**) Mean bioluminescent signal for Probe 1 in primary fibroblasts (**A–C**,**E**–**H**) or iPSC-derived neurons (**D**) from control individuals and *PARK2*/*PINK1* patients (**A**), a subset of or single *PARK2* patients (**B**–**D**,**G**,**H**), or *PINK1* patients (**E**–**H**), showing a weaker signal in *PINK1* patients and *PARK2* Patients 1-3, associated with presence of the partially functional Parkin R42P substitution, and rescue effects of Parkin, PINK1, PINK1 E240K or Ub S65D/A overexpression. The numbers on the graphs indicate the mean signals for individual patients, as presented in Supplementary Table 1, with *n* = 4–6 independent experiments (**A**,**B**,**E**), *n* = *4–5* wells from one experiment representative of three (**C**,**F**), or *n* = 4–10 wells from one of 2–5 independent experiments (**D**,**G**,**H**). Note that the overproduction of Parkin, PINK1 or phosphomimetic Ub S65D partially normalizes the signal in fibroblasts from individual *PARK2* or *PINK1* patients, whereas PINK1 E240K and Ub S65A do not have any effect. Results are expressed as means ± SEM. *p < 0.05, **p < 0.01, ***p < 0.001 versus control individuals (**A**,**B**,**D**,**E**, Student’s *t*-test) or the conditions indicated (**C**,**F**–**H**, one-way ANOVA with Dunnett’s (**C,F**) or Holm-Sidak’s (**G**,**H**) posthoc tests).

Finally, we investigated mitochondrial import efficiency comparatively in primary fibroblasts from PD patients with *PARK2* (n = 7) or *PINK1* (n = 2) mutations, and from control individuals (n = 6; Supplementary Table 1). Transfection efficiencies were lower in fibroblasts than in HEK293T cells, such that Probe 2 was barely detectable in these cells. We therefore used Probe 1, for which a consistent signal was obtained, with no toxicity in the experimental conditions used (Supplementary Fig. 5A and B). The mean intensity of the Probe 1 bioluminescent signal tended to be lower in fibroblasts from *PARK2*/*PINK1* patients than in those from control individuals, although this difference was not statistically significant (−25%, Fig. 5A). In cells from five patients, the signal was 40% to 75% lower than the mean signal in control cells (Fig. 5B,E). These patients carried either a homozygous *PINK1* mutation leading to the Q456* substitution associated with a kinase-dead protein^53^ (patients 8 and 9, Supplementary Table 1 and Fig. 5E) or compound *PARK2* heterozygous mutations associated with an inactive truncated protein (allele 1) and the Parkin A42P variant, which relieves UBL-mediated autoinhibition and preserves enzymatic activity (allele 2; patients 1-3, Supplementary Table 1 and Fig. 5B)^34,35,54^. The other patients with *PARK2* mutations (patients 4-7) carried inactivating deletions or missense mutations, suggesting that an abnormal protein with residual activity may be more deleterious than an inactive protein. Similarly, induced-pluripotent stem cell (iPSC)-derived neurons from patient 2 showed a 40% decrease in Probe 1 signal intensity compared to *in vitro* differentiated neurons from a control individual (Fig. 5D and Supplementary Fig. 5C). Overexpression of Parkin increased the signal intensity in fibroblasts from both *PARK2* patients 3 and 5 (Fig. 5C), whereas overexpression of PINK1 only tended to rescue the signal in fibroblasts from *PINK1* patient 9 (Fig. 5F). As expected, the kinase-dead PINK1 variant E240K did not have any effect. Unlike Parkin, PINK1 and the probe are both imported through the TOM complex and may thus compete with each other for import, thereby explaining why PINK1 overexpression cannot fully rescue the signal in cells devoid of functional PINK1. We hypothesized that phosphomimetic Ub S65D might enhance Parkin activity in cells from patients with residual enzymatic activity, or bypass the requirement for PINK1 for Parkin activation in cells from patients with dysfunctional PINK1. Consistent with this possibility, the bioluminescence signal of Probe 1 was rescued in fibroblasts from both *PARK2* patient 3 and *PINK1* patient 9 by overproduction of Ub S65D (+150% versus that in the absence of Ub S65D), reaching values similar to those obtained for Control 1 in the absence of Ub S65D (Fig. 5G). In addition, Ub S65D overexpression nearly doubled the signal in Control 1, consistent with our observations in HEK293T cells (Fig. 4B). In contrast, as expected, Ub S65D had no effect in *PARK2* patient 5, who carries two loss-of-function *PARK2* alleles. In addition, there was no change in the intensity of the bioluminescence signal in cells from patients 3, 5 and 9 following overexpression of the non-phosphorylatable Ub S65A variant (Fig. 5H).

## Discussion

**Figure 6:**
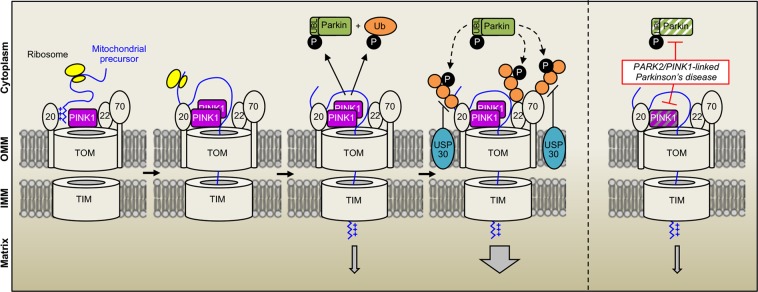
Model illustrating facilitation of mitochondrial protein import by the PINK1 kinase-driven E3 Ub ligase Parkin and lack thereof in *PARK2*/*PINK1*-linked Parkinson’s disease. Under physiological reductions in mitochondrial protein import efficiency, the PINK1 kinase associates with the TOM complex and activates Parkin by phosphorylating S65 of Parkin and ubiquitin. By ubiquitylating receptor subunits of the TOM complex, Parkin facilitates the import of proteins targeted to mitochondria by the presequence pathway. The mitochondrial ubiquitylase USP30 antagonizes these effects. Mutations in *PINK1* or *PARK2* impair the system and its regulatory effect on mitochondrial protein import, contributing to the pathophysiology of autosomal recessive PD.

Recent studies have highlighted the remarkable plasticity of the mitochondrial protein import process in response to metabolic requirements and stress^55^, but our knowledge of the cytosolic mechanisms regulating mitochondrial import in mammalian cells remains poor. We here showed that one of the major mitochondrial protein import pathways, the presequence pathway, is facilitated by the PINK1 kinase-driven Parkin Ub ligase and antagonized by the ubiquitylase USP30 (Fig. 6). Previous studies in yeast have identified the ubiquitin-proteasome degradation system as a constitutively active negative regulator of the biogenesis of intermembrane space proteins through competition with their mitochondrial import^56^. Our findings reveal another level of mitochondrial import regulation by Ub that presumably optimizes this process to physiological requirements.

Unlike other functions exerted by the PINK1/Parkin system in mitochondrial quality control^9,57^, its effect on import was observed in cells that were not treated with mitochondrial toxins, in the absence of gross alterations in Δψ_mit_, suggesting a role in response to physiological fluctuations in import efficiency. Such a situation, mimicked here artificially by the overexpression of a probe targeted to mitochondria, may be associated with mild local reductions in Δψ_mit_ driving activation of the PINK1/Parkin system. In cells expressing the probe, we observed a pattern of Parkin-dependent ubiquitylation at the OMM that was different from that built up following massive mitochondrial depolarization, associated with an increase in K11 Ub chains. Parkin has been reported to assemble both canonical Ub chains, through K48 and K63, and noncanonical chains, involving K6 and K11 on depolarized mitochondria^35,37,58^, with a greater relative increase for K48 and K63 chains^35,58^. Our observations indicate that the assembly of K11 chains may represent an attempt to restore the mitochondrial import process.

Whether post-translational modifications of subunits of the TOM complex affect mitochondrial import efficiency in mammalian cells is unknown. To date, the phosphorylation by cytosolic kinases is the only modification reported to modulate the activity of the TOM machinery in yeast^55,59^. We found that mitochondrial import facilitation by the PINK1/Parkin system was accompanied by ubiquitylation of TOM20, TOM70, and TOM22, which were also modified following treatment with AO. Other proteins modified by the PINK1/Parkin system on severely damaged mitochondria (CISD1, Mfn2) were not ubiquitylated in our paradigm. These findings raise the possibility that, depending on the type of chains formed, Ub modification of the TOM machinery may either precede its degradation in the context of mitophagy^31,37,38,51^ or affect import efficiency.

Future studies will need to clarify the precise mechanisms by which ubiquitylation regulates mitochondrial import through the TOM/TIM complexes, the type of modification involved and the physiological conditions under which it occurs. Such mechanisms may involve the major receptor subunits of the TOM complex, which are among the relatively few proteins to be regulated antagonistically by both Parkin and USP30^37,51^. TOM70 is only loosely and transiently associated with the core structure of the TOM complex^60^ and may thus be a preferential target for posttranslational regulatory mechanisms. We and others have shown that Parkin physically associates with the import receptor TOM70^17,31,50^ and a number of Parkin-dependent ubiquitylation sites have been identified in this TOM subunit^50^. Here, TOM70 silencing enhanced mitochondrial import through the TOM20-dependent pathway to a similar extent to activation of the PINK1/Parkin system. It is, thus, conceivable that the ubiquitin-dependent degradation of TOM70 allows for optimization of the translocation of proteins with classical MTS across the TOM40 channel. Subsets of TOM complexes with different relative ratios of TOM70 and TOM40 have been identified by size exclusion chromatography^26^ and may possibly transport different categories of proteins.

As an alternative or complementary mechanism, the PINK1/Parkin system may stimulate import by targeting the preprotein receptor TOM20. Notably, the recently described role of PINK1 and Parkin in the translational derepression of specific RCCs occurs through a combination of mechanisms involving TOM20 and other factors^16^. Physical association between PINK1 and TOM20 was found to be essential to anchor RCC mRNAs to the OMM and to promote cotranslational import of the encoded proteins through the TOM complex. It remains unclear whether this mechanism involves ubiquitylation by Parkin, but it is tempting to speculate that it plays a more general role in facilitating mitochondrial import. Cotranslational import mediated by the localization of mRNAs close to the OMM has indeed emerged as a functionally conserved phenomenon of broader relevance than initially suspected^16,61–65^. More than half the mRNAs encoding mitochondrial proteins are located in the vicinity of the mitochondria in yeast^56,57,59,66–68^, and TOM20 and the MTS of the nascent protein, two essential components of the presequence import pathway, play a key role in mediating this association both in yeast and in mammalian cells^16,61–65^.

In conclusion, through the development of a new chemogenetic tool, we identified a cooperative role of PINK1 and Parkin in the regulation of mitochondrial protein import, thereby broadening our understanding of the mechanisms by which these proteins regulate mitochondrial quality control. Specific *PINK1* and *PARK2* mutations were associated with reductions in mitochondrial import efficiency in cells from patients with autosomal recessive PD, underpinning the pathophysiological relevance of these findings. Such a mechanism may play a more general role in PD, following environmental and age-associated inactivating modifications of the PINK/Parkin system^69–71^. Together with the recent implication of α-synuclein in mitochondrial import blockade in PD^8^, our study suggests that defective protein transport through the TOM complex may be a unifying pathway to dopaminergic neuronal degeneration.

## Methods

### Human cells

Human skin fibroblasts were obtained from PD patients and healthy individuals by routine clinical procedures at the Clinical Investigation Center for Neurosciences at the Pitié-Salpêtrière Hospital, and were reprogrammed into iPSC and differentiated into dopaminergic neurons as previously described^72^. All patients and healthy individuals signed and informed consent before the procedure. The study was approved by a local ethics committee (CCPPRB du Groupe Hospitalier Pitié-Salpêtrière, Paris, France), promoted by INSERM and performed in accordance with guidelines established by the World Medical Association Declaration of Helsinki.

### Probes, plasmids, siRNAs and lentiviral vectors

The cDNAs corresponding to Probes 1-3 were obtained by DNA synthesis (Genecust, Dudelange, Luxembourg) as fusions of modules encoding the following protein/protein domains (Fig. 1A): the MTS of human DLD (NP_001276681; amino acids 1 to 36; Probes 1 and 2); RGFP (AF372525; aa 2 to 233; Probes 1-3); a linker region including a cMyc-tag (EEQKLISEEDLGIPPARAT; Probes 1-3); RLuc (P27652; amino acids 1 to 311; Probes 1-3); a second linker (GGGGSGGGGS; probes 1-3); the human FKBP1A DD domain^22^ (NP_000792; amino acids 2 to 108 with mutations encoding E32G F37V R72G K106E substitutions; Probes 1-3); the mouse ornithine decarboxylase PEST domains^23,24^ (NP_038622; aa 422 to 461; Probe 2). The cDNAs were inserted into the pCl-neo mammalian expression vector (Promega, Madison WI) between the *Nhe*I and *Not*I sites. The other plasmids used were: pcDNA3.1 containing sequences encoding human HA-Parkin or HA-Parkin variants^32^; HA-Parkin W403A; HA-Parkin and HA-Parkin-S65A/E^73,74^ (kindly provided by N. Matsuda); pCMV(d1)-PINK1 (WT)-3HA and pCMV(d1)-PINK1 E240K-3HA^13,75^ (kindly provided by N. Matsuda); V5-HSD17B10^17^; pEF-HA-Ub K0 (kindly provided by Y. Yarden) and pCDNA3.1 encoding Flag-Ub S65A/D^13^ (kindly provided by N. Matsuda); pCAGGS containing sequences encoding human Flag-USP30 and Flag-USP30-C77A (kindly provided by B. Bingol)^51^. The following siRNAs were used at a final concentration of 20 nM: AllStars negative control siRNA (1027281) and validated TOM70 (SI00301973), TOM20 (SI00301959), TIM23 (SI05217905)-specific siRNA (Qiagen), MPPB (L-004747-00-0005)-specific siRNA (Dharmacon), PGC-1-α siRNA (sc38884, Santa Cruz), RNAi negative control (12935-200), PINK1 (HSS127945) and Parkin (HSS107593)-specific stealth siRNA (Thermo Fisher). Lentiviral particles expressing Probe 1 were produced by transient cotransfection of HEK293T/17 cells (ATCC No. CRL-11268) with a lentiviral recombinant vector carrying the transgene of interest, an encapsidation plasmid (p8.9) and a VSV envelope expressing plasmid (pVSV-G), as previously described^76^. Lentiviral particles titers were determined by quantitative PCR (qPCR)^77^.

### Cell culture, transfection, lentiviral infection, and pharmacological treatments

Cells were cultured in DMEM-GlutaMAX medium with glucose at 4.5 g/L (Thermo Fisher) supplemented with FBS (Thermo Fisher) and 1% penicillin/streptomycin (Thermo Fisher), unless otherwise stated. HEK293T cells were transfected in the presence of Lipofectamine 2000 (Thermo Fisher), according to the manufacturer’s instructions. Inducible HeLa Flp-In T-REx (HFT) cells with or without expression of PARKIN^WT^ or PARKIN^C431S^ proteins, or lacking PINK1 were previously described^35^. To induce each protein of interest, cells were treated with 0.5 μM doxycycline (DOX) for 16 hr. Cells were depolarized with a mixture of antimycin A (10 mM) and oligomycin A (5 mM) (Sigma) for the indicated time periods. Fibroblasts were transfected by electroporation with a Neon^TM^ Transfection System (Invitrogen), according to the manufacturer’s instructions. In general, co-transfections were performed using a weight ratio of 2:1 between the vector encoding Probe 2 and vectors encoding any other protein of interest. In primary human fibroblasts, the weight ratio between the vector encoding Probe 1 vector and vectors encoding Flag-Ub S65A/D was 1:0.125. Where indicated, cells were incubated with 10 µM CCCP (Sigma) for 24 h; 50 nM Bafilomycin A (AG Scientific) for 2 h; 100 mM epoxomicin (Sigma) for 24 h; 100 µM 3-methyladenine (Sigma) for 6 h; 10 µg/µl E-64d (Sigma) for 2 h; 0.5 µM fresh Shield1 (Clontech) for 7 h or 24 h; 5 µM MG132 (Sigma) for 7 h; or 5 µM (HEK293T) or 25 µM (primary fibroblasts) CLZ400A immediately before bioluminescence measurement. Where indicated, living cells were incubated with mitochondrial dyes (Invitrogen) at a concentration of 10 nM (TMRM, T668) or 200 nM (MitoTracker Deep Red), at 37 °C for 30 minutes. Infections of iPSC-derived neurons^72^ with the lentivirus vector encoding Probe 1 were carried out on day 28 of differentiation at a multiplicity of infection of 10 and the bioluminescent signal was measured on day 35.

### Fluorescence microscopy and bioluminescence assay

Images of probe expression and colocalization with mitochondrial dyes were obtained with an inverted spinning disk microscope from Till photonics fitted with an Olympus 100x oil immersion objective (N.A 1.4). Probe induction was analyzed with ImageJ software, on fluorescence images from at least 15 cells per condition. For bioluminescence assays, cells were plated in black precoated 96-well plates (Greiner Bio-One), at a density of 25,000 (HEK293T) or 10,000 cells/well (primary fibroblasts). They were analyzed 48 h after transfection in Hank’s Balanced Salt Solution (Invitrogen) containing 20 mM HEPES (Invitrogen), with an FDSS 7000 Functional Drug Screening System (Hamamatsu). A control condition with CCCP treatment was systematically included in each experiment. Bioluminescence signals were collected every second for five minutes after the addition of CLZ400A to the cells, and normalized relative to the total RGFP fluorescence of the probe in each well, recorded at an excitation wavelength of 470 nm, and an emission wavelength of 510 nm. For Supplementary Fig. 2C, bioluminescence signals were also normalized relative to the intensity of MitoTracker Deep Red staining, captured with the Odyssey Imaging (Li-COR) system and quantified with Li-COR Image Studio. For Fig. 5D, bioluminescence signals were normalized relative to the intensity of anti-GFP immunostaining signal, measured with an Arrayscan XTi automated fluorescence microscope (ThermoScientific, Courtaboeuf, France). On graphical representations of bioluminescence peaks, each dot corresponds to the mean peak value for three to eight wells in a single independent experiment, determined as follows: for each well, the peak value was obtained as the mean of the bioluminescence signals recorded during the first 100 seconds normalized against fluorescence, corrected by subtracting the mean signal obtained for cells treated with CCCP (calculated from data for 3–8 wells). In graphical representations of the results for fibroblasts, each number/patient corresponds to the mean peak bioluminescence signal from four to six independent experiments, with six wells per experiment, collected as indicated above.

### Subcellular and submitochondrial fractionation, mitochondrial import assays and western blotting

Total protein fractions were obtained from cells lysed at 4 °C for 30 minutes in RIPA buffer (50 mM Tris-HCl, 150 mM NaCl, 1% Triton, 0.5% sodium deoxycholate, 0.1% SDS, 1 mM EDTA, pH7.4) or in 50 mM Tris-HCl, 150 mM NaCl, 1% Triton, 1.5 mM MgCl_2_, 1 mM DTT, pH 7.4 supplemented with phosphatase and protease inhibitors (Complete Cocktail, Thermo Fisher), and centrifuged at 16,100 × g. Mitochondrion-enriched fractions were obtained by differential centrifugation, from cells homogenized with a Dounce homogenizer (80 manual strokes)^17^ **(**HEK293T), or as described in^35^ (HeLa Flp-In T-REx). They were then subjected to digestion with trypsin (Sigma, 1 µg/mg of protein) for 20 minutes or with 50 µg/ml proteinase K (PK) for 15 min on ice with or without prior swelling in 20 mM HEPES-KOH pH 7.6. PK digestion of mitochondrial proteins was stopped with 2 mM PMSF. Where indicated, 1% (v/v) Triton X-100 was added prior to PK treatment. Samples were precipitated by 15% TCA (trichloric acid) on ice for 10 minutes.

For mitochondrial import assays (Supplementary Methods), Probe 2 was transcribed and translated *in vitro* using the TNT-coupled reticulocyte lysate system (Promega) following manufacturer instructions, in presence or absence of 0.5 µM Shield1.

Where indicated, proteins were analyzed by SDS-PAGE and western blotting with the antibodies indicated in Supplementary Table 2. Proteins were visualized with fluorescent antibodies or by enhanced chemiluminescence (Pierce). Fluorescence/chemiluminescence signals were captured with the Odyssey Imaging (Li-COR) system, detected on a film (ECL, Amersham Hyperfilm) or with the Chemidoc touch Imaging System (Bio-Rad) and quantified with Li-COR Image Studio, Image Lab Software (Bio-Rad) or ImageJ software (NIH). The blots were cropped for improved clarity and full-length blots were included in the Supplementary Informations.

### Mitochondrial poly-ubiquitin capture and UB-AQUA/PRM proteomics

Mitochondrially-derived ubiquitylated proteins were purified using Halo-4xUBA^UBQLN1^, as described^35^. Briefly, crude mitochondrial extracts (100 μg) were incubated at 4 °C for 3 hours with 10 μl of Halo-4xUBA^UBQLN1^ beads (pack volume). Following 4 washes with lysis buffer containing 1 M NaCl and one final wash in 10 mM Tris pH 8.0, proteins were eluted using sample buffer prior to analysis. For UB-AQUA/PRM proteomics (Supplementary Methods), the mitochondrion-enriched fractions were lysed in 50 mM Tris/HCl pH 7.5, 1 mM EDTA, 1 mM EGTA, 50 mM NaF, 5 mM sodium pyrophosphate, 10 mM sodium 2-glycerol 1-phosphate, 1 mM sodium orthovanadate, 1% (v/v) NP-40, 1 mg/ml aprotinin, 1 mg/ml leupeptin, 1 mM benzamidine, 1 mM AEBSF, 10 mM PR-619, 50 mM chloroacetamide and 1x PhosSTOP phosphatase inhibitor Cocktail (Roche). The fractions were then sonicated and clarified by centrifugation (16,000 x g for 10 min at 4 °C). Protein concentrations were determined by the Bradford assay. Proteins (20 µg) were precipitated with TCA. Samples were digested first with Lys-C [in 100 mM tetraethylammonium bromide (TEAB), 0.1% Rapigest (Waters Corporation)] for 2 hours at 37 °C, followed by the addition of trypsin and further digested for 6 hours at 37 °C. Digests were acidified with an equal volume of 5% (vol/vol) formic acid (FA) to a pH of ~2 for 30 min, dried down, and resuspended in 1% (vol/vol) FA.

### Quantitative real-time RT-PCR

Total RNA was purified with the RNeasy plus Mini Kit (Qiagen) and reverse-transcribed (1 µg) to generate cDNA (iScript cDNA Synthesis, Bio-Rad). Real-time PCR was performed with the primers indicated in Supplementary Table 3, SsoAdvanced Universal SYBR Green Supermix (Bio-Rad) and the LightCycler 480 System (Roche Applied Science). Results were analyzed with LightCycler 480 sw 1.5 quantification software. The data for each gene of interest were normalized relative to *ACTB* expression.

## Supplementary information

Supplementary Informations
