# Supplementary material for: The PINK1 kinase-driven ubiquitin ligase Parkin promotes mitochondrial protein import through the presequence pathway in living cells
Source: Sci Rep. 2019 Aug 14;9:11829. doi: 10.1038/s41598-019-47352-9 (PMC6694185; doi:10.1038/s41598-019-47352-9)
Supplement: Supplementary file 1 — Supplementary Informations [file 41598_2019_47352_MOESM1_ESM.pdf]

The PINK1 kinase-driven ubiquitin ligase Parkin promotes mitochondrial protein import through the presequence pathway in living cells

M. Jacoupy, E. Hamon-Keromen, A. Ordureau, Z. Erpapazoglou, F. Coge, J-C. Corvol, O. Nosjean, C. Mannoury La Cour, M. J. Millan, J. A. Boutin, J. W. Harper, A. Brice, D. Guedin, C. A. Gautier, O. Corti

Supplementary Legends

Supplementary Table 1. Characteristics of the donors of the primary skin fibroblasts used in the study.

Supplementary Table 2. Antibodies used for western blotting.

Supplementary Table 3. Sequence of primers used for quantitative RT-PCR analysis.

Supplementary Fig. 1. Validation of Probe 2 in HEK293T cells.

Supplementary Fig. 2. Parkin modulates the mitochondrial presequence import pathway in HEK293T cells.

Supplementary Fig. 3. The stimulation of mitochondrial import by Parkin is PINK1 kinase- and ubiquitin-dependent.

Supplementary Fig. 4. Parkin modulates mitochondrial import through its E3 ubiquitin-protein ligase activity.

Supplementary Fig. 5. Validation of Probe 1 in human primary fibroblasts (Control 1) and in iPSC-derived neurons.

Supplementary Fig. 1. Validation of Probe 2 in HEK293T cells.

Representative western blots and corresponding quantifications, illustrating (**A**) the localization of Probe 2 in trypsin-digested mitochondrion-enriched fractions from cells treated with Shield1 and the absence of a signal following CCCP treatment; and (**B**) stabilization of the probe by Shield1 and proteasome inhibition (MG132) in total cell fractions. Loading controls: β subunit of MPP (MPPβ) and α-tubulin. (**C**) Western blot analyses of total cell fractions, illustrating the downregulation of molecular components of mitochondrial import pathways by specific siRNAs. (**D**) Silencing of TOM20, TOM70, TIM23 and MPPβ does not affect the abundance of other markers of mitochondrial subcompartments (Mfn2: mitochondrial outer membrane, ATP5a1: mitochondrial inner membrane, HSD17B10: mitochondrial matrix). (**E**) Representative western blot showing the gradual increase of HSD17B10 expression analyzed in Fig. 3F. Loading control: α-tubulin. (**F**) Input (20%) and *in vitro* mitochondrial import assay, showing that Probe 2 is imported into mitochondria and stabilized by Shield1. PK = Proteinase K. Results are expressed as means ± SEM, with n = 3 independent experiments. Data were analyzed by one-way (**D**) or two-way ANOVA (**A, B**) with Dunnet's (**B, D**) or Holm-Sidak's (**A**) post-hoc tests. *p<0.05, **p<0.01, ***p<0.001.

Supplementary Fig. 2. Parkin modulates the mitochondrial presequence import pathway in HEK293T cells.

(**A**) Comparative quantitative analysis of the bioluminescent signals of Probe 2 in cells overproducing Parkin or transfected with empty pcDNA (signals arbitrarily set at 100%) after 3, 8 or 24 h of treatment by Shield1. n = 4 independent experiments (**B**) Efficiency of *PARK2* and *PINK1* silencing with specific siRNAs, as shown by quantitative RT-PCR. n = 3 independent experiments. (**C**) BRET signal of Probe 2 in control cells or cells coproducing Parkin, normalized against probe fluorescence (left graph) or MitoTracker Deep Red staining intensity (right graph). n = 6 wells from one representative experiment. (**D-G**) Western blot analyses of total cell fractions, illustrating the overproduction or the silencing of each protein of interest in the experiments presented in Fig. 3. Loading control: α-tubulin. Results are expressed as means ± SEM. Data were analyzed with Student's t test (**A, B**) or two-way repeated-measures ANOVA with Dunnet's post-hoc test (**C**). *p<0.05, **p<0.01, ***p<0.001.

Supplementary Fig. 3. The stimulation of mitochondrial import by Parkin is PINK1 kinase- and ubiquitin-dependent.

(**A-G**) Western blot analyses of total cell fractions, illustrating the overproduction of each protein of interest in the experiments presented in Fig. 4. Loading control: α-tubulin.

Supplementary Fig. 4. Parkin modulates mitochondrial import through its E3 ubiquitin-protein ligase activity.

(**A**) Quantitative analysis of the relative bioluminescent signals for Probe 2 in HeLa Flp-In T-Rex cells treated or not with doxycycline (dox) in order to overproduce Parkin or Parkin C431S. $n = 3$ independent experiments. Results are expressed as means ± SEM. Data were analyzed with Student's *t* test (**A**). $^{**}p<0.01$, $^{***}p<0.001$ (**B**) Representative western blot showing Parkin or Parkin C431S expression after induction with doxycycline. §: Residual band from anti-Parkin immunoblotting. (**C**) Western blot analysis of ubiquitylated proteins pulled down with TUBEs from mitochondrion-enriched fractions isolated from cells in the conditions illustrated in Fig. 4F, using antibodies against ubiquitin, MFN2 and CISD1. (**D**) Quantification by UB-AQUA proteomics of p-S65 ubiquitin chain associated with mitochondria in HeLa Flp-In T-REx $Parkin^{WT}$ or $Parkin^{C431S}$ cells depleted or not of PINK1 and expressing Probe 2 in the presence of Shield1 (P+S), or treated with S or AO. (**E-F**) Western blot analysis of total cell fractions from the experiment shown in Fig. 4G, illustrating the overproduction of each protein of interest.

Supplementary Fig. 5. Validation of Probe 1 in human primary fibroblasts (Control 1) and in iPSC-derived neurons.

(**A**) Quantitative analysis of BRET signals in cells with and without Shield1 and/or CCCP (24 h) treatment, following the addition of CLZ400A. $n = 8$ wells from one representative experiment. (**B**) Representative fluorescence images and higher magnifications (framed regions in the overlay) showing the colocalization of Probe 1 (RGFP signal) and TMRM signals in living cells treated for 24 h with Shield in fibroblasts from a control individual (Control 1) and a *PARK2* patient (Patient 3). (**C**) Representative fluorescence images and higher magnifications (framed regions in the

overlay) showing the colocalization of Probe 1 (RGFP signal) and Tubulin beta-3 chain (TuJ1) signals in induced-Pluripotent Stem Cell (iPSC)-derived neurons from Control 7 (Supplementary Table 1) treated for 24 h with Shield. Scale bar: 10 µm. Results are expressed as means ± SEM. **$p<0.01$ versus vehicle/vehicle; ##$p<0.01$ versus Shield1/CCCP (**A**, two-way repeated-measures ANOVA with Tukey's post-hoc test).

Supplementary Fig. 1

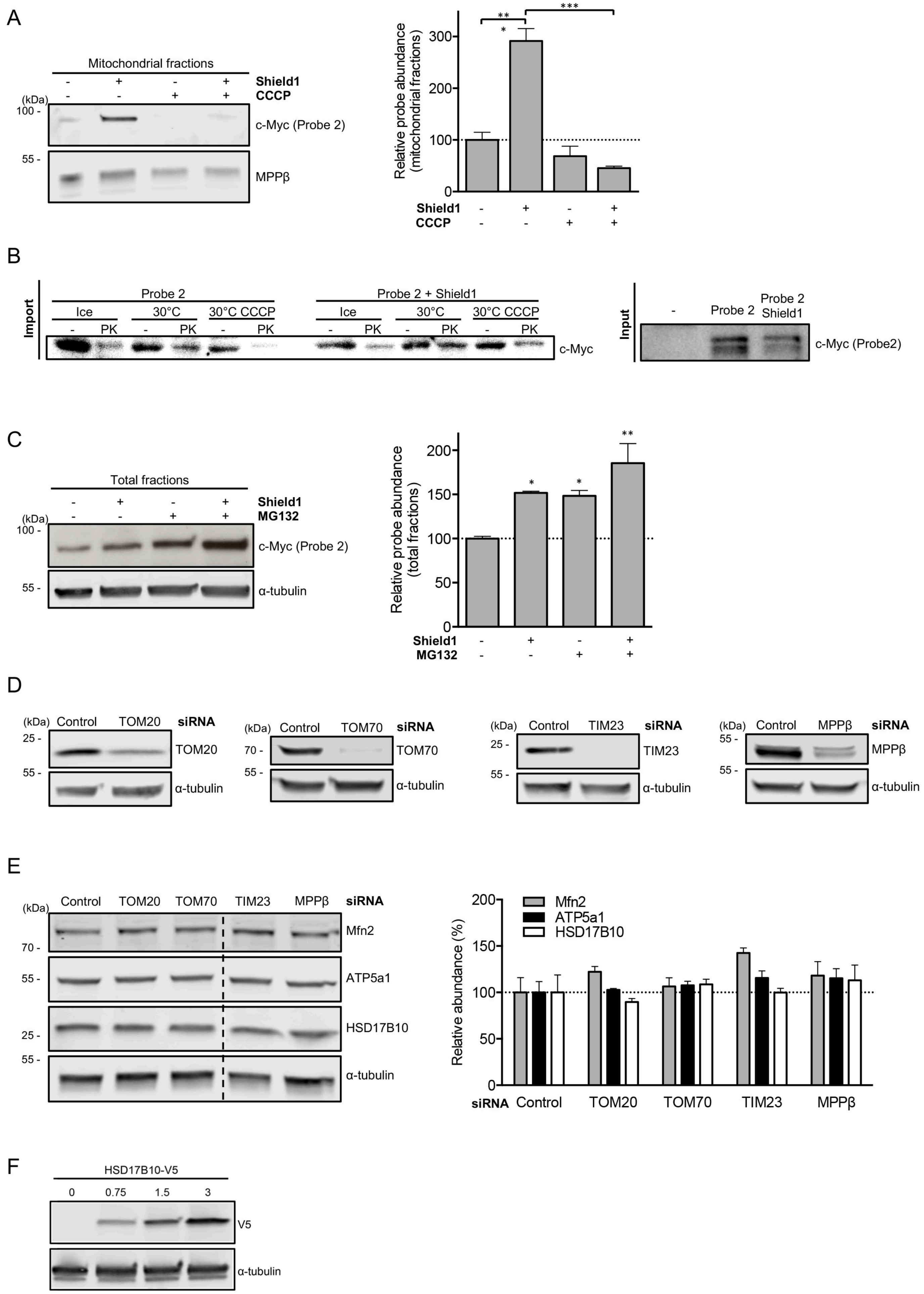

Supplementary Fig. 2

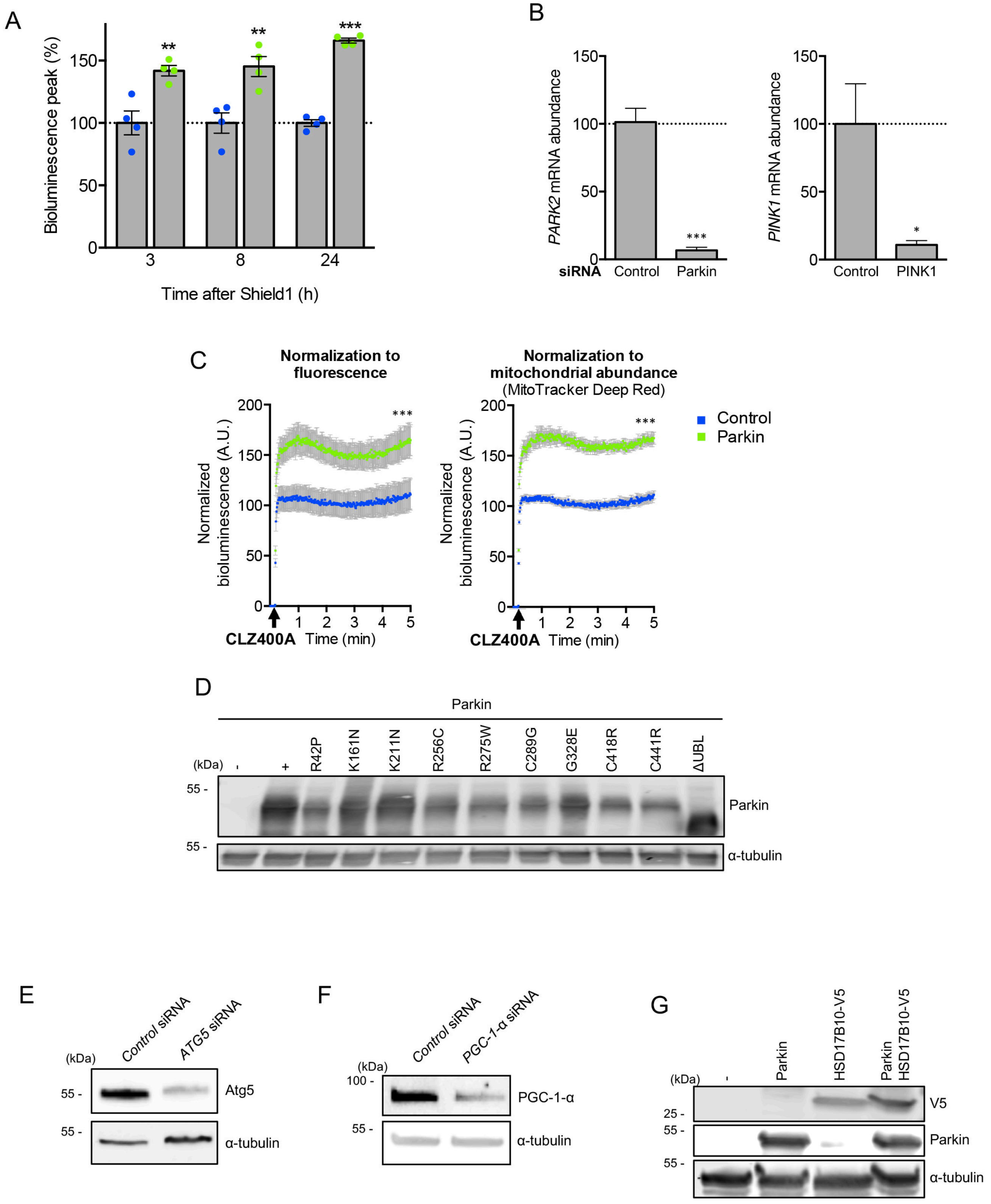

Supplementary Fig. 3

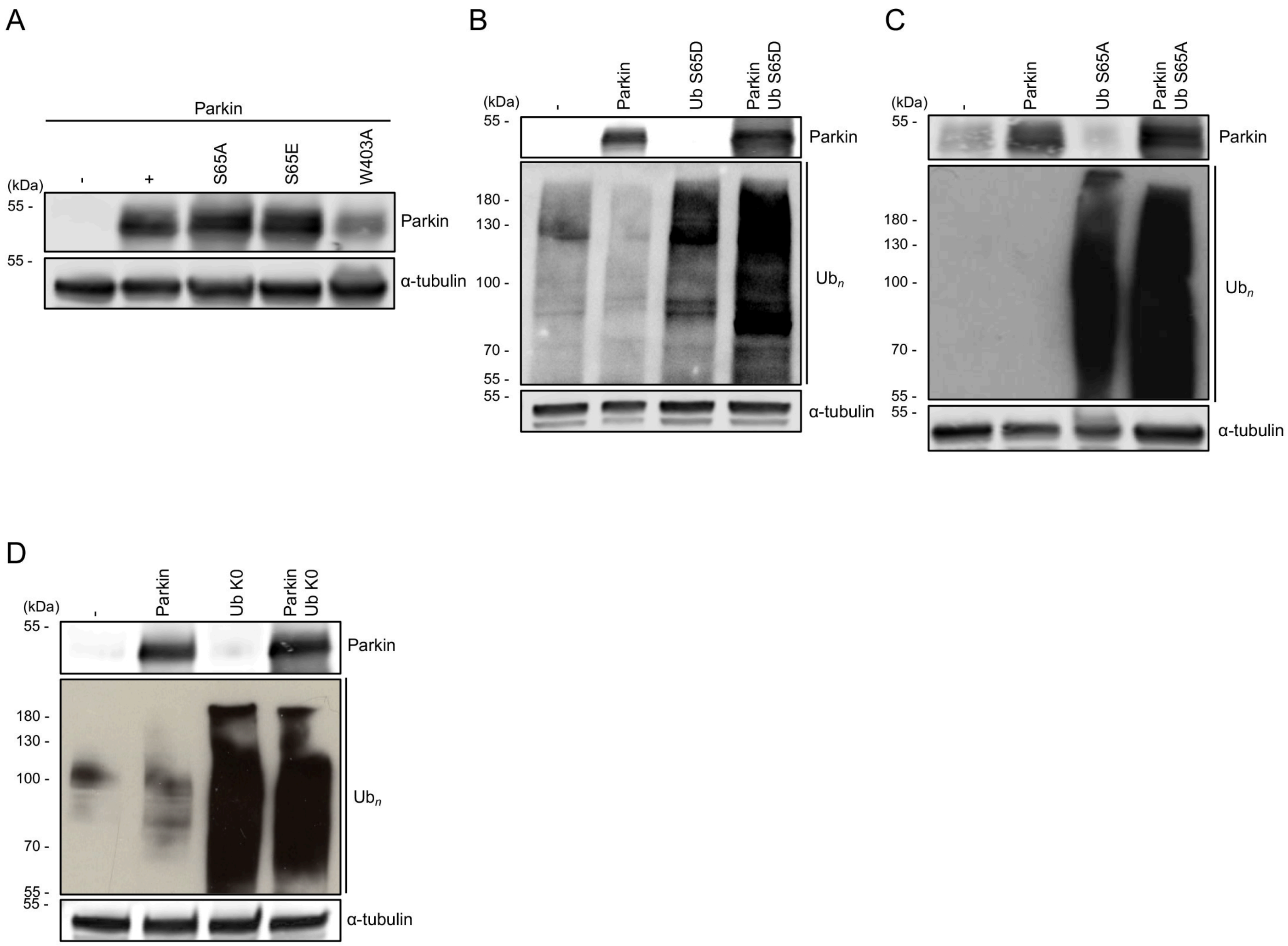

Supplementary Fig. 4

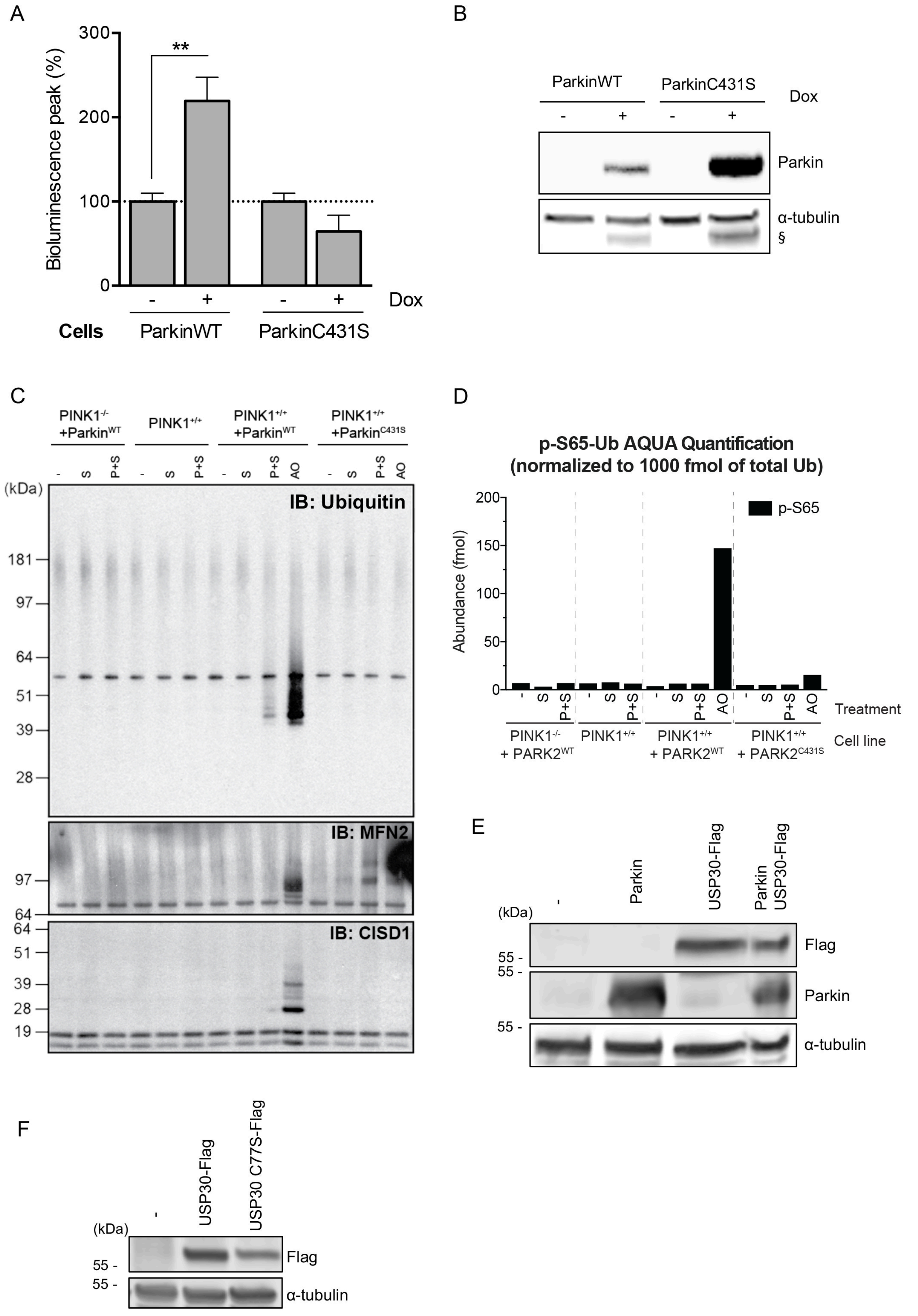

Supplementary Fig. 5

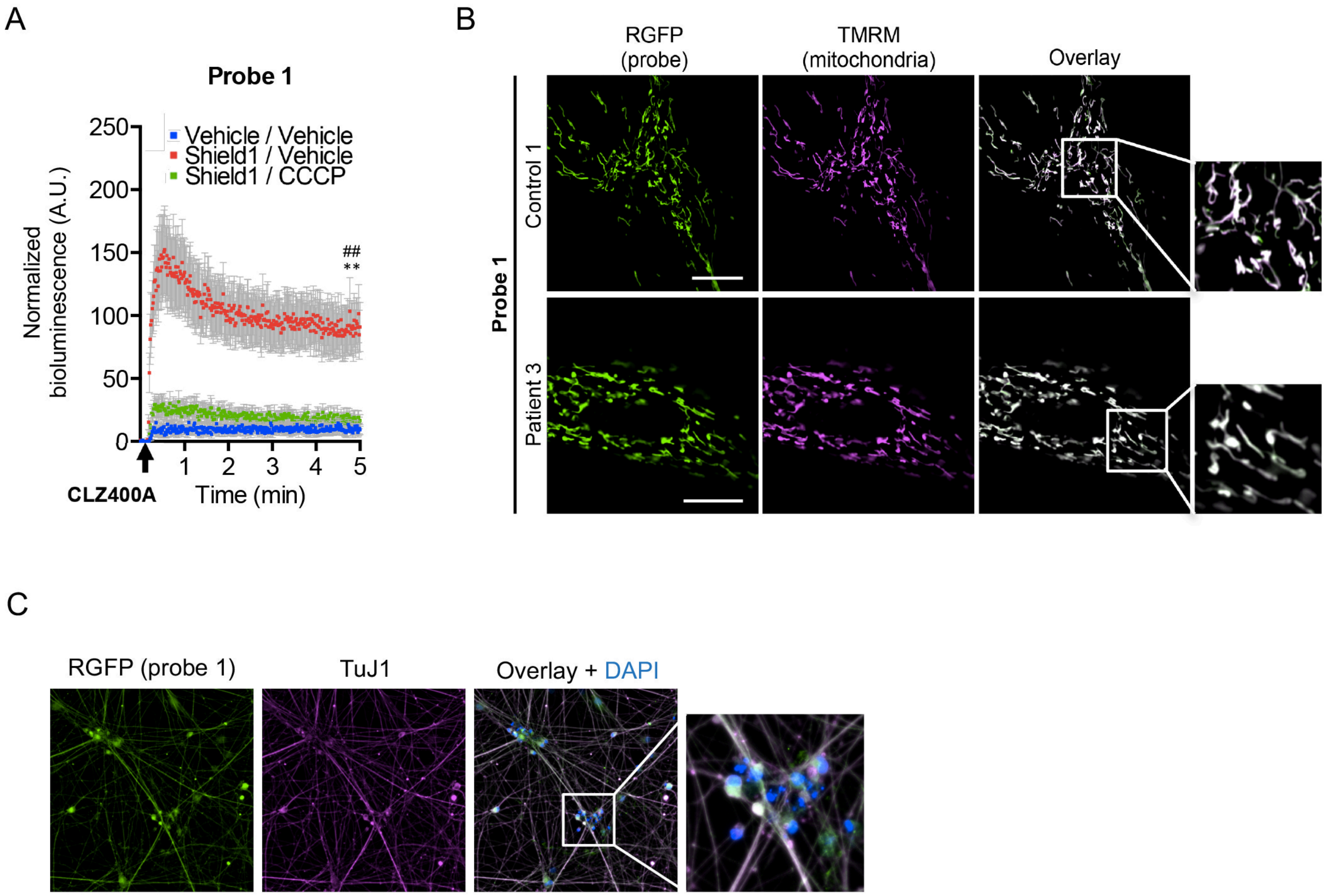

Figure 2D

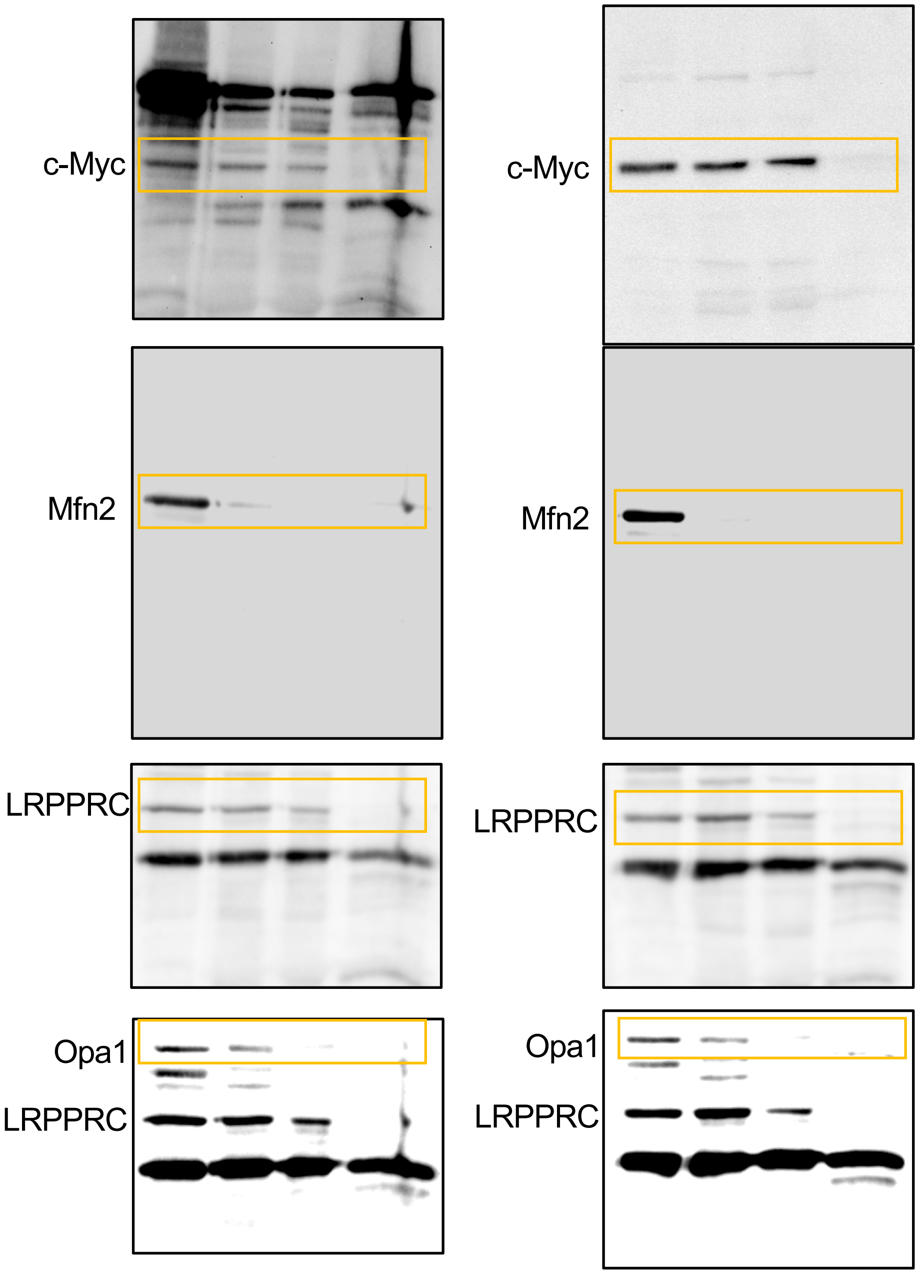

Figure 4F

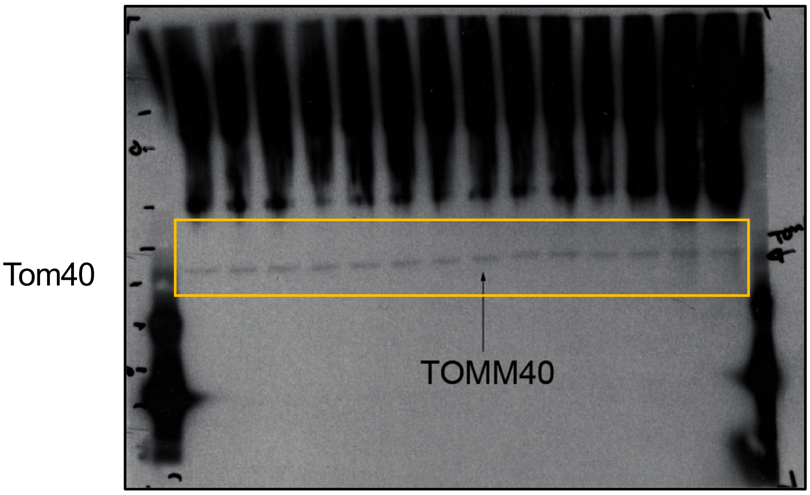

Figure S1A

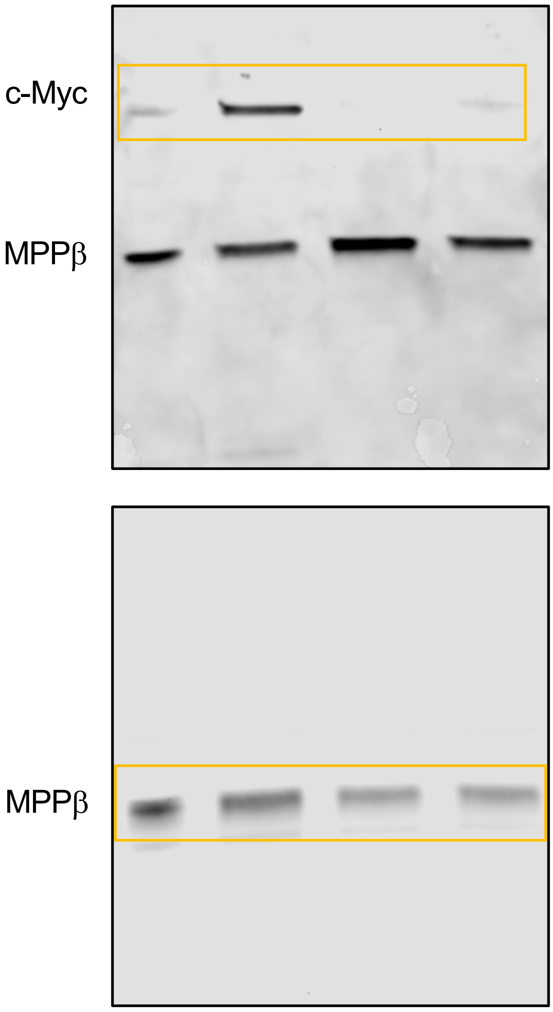

Figure S1C

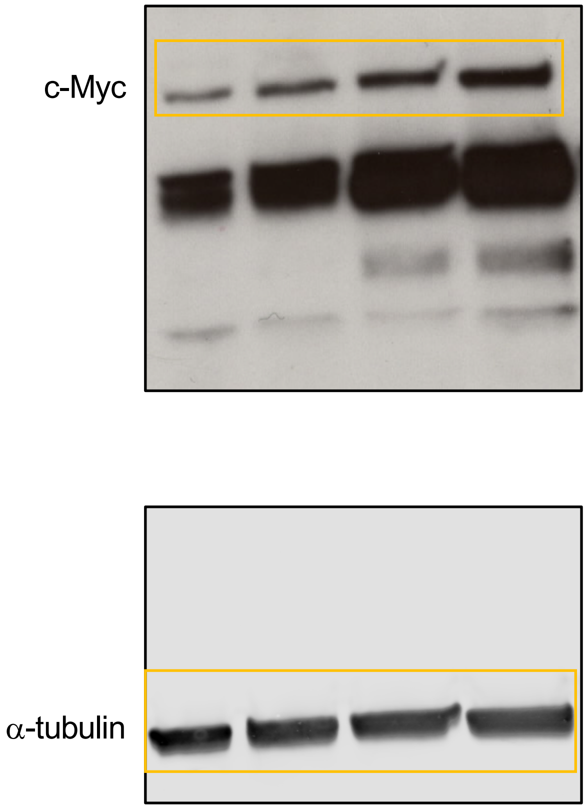

Figure S1B

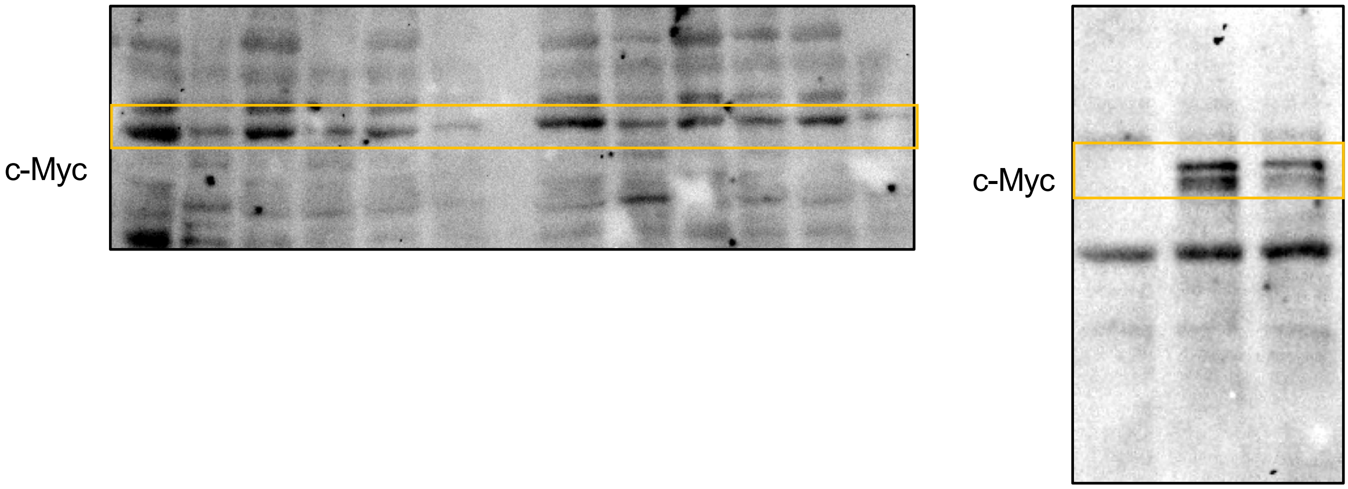

Figure S1D

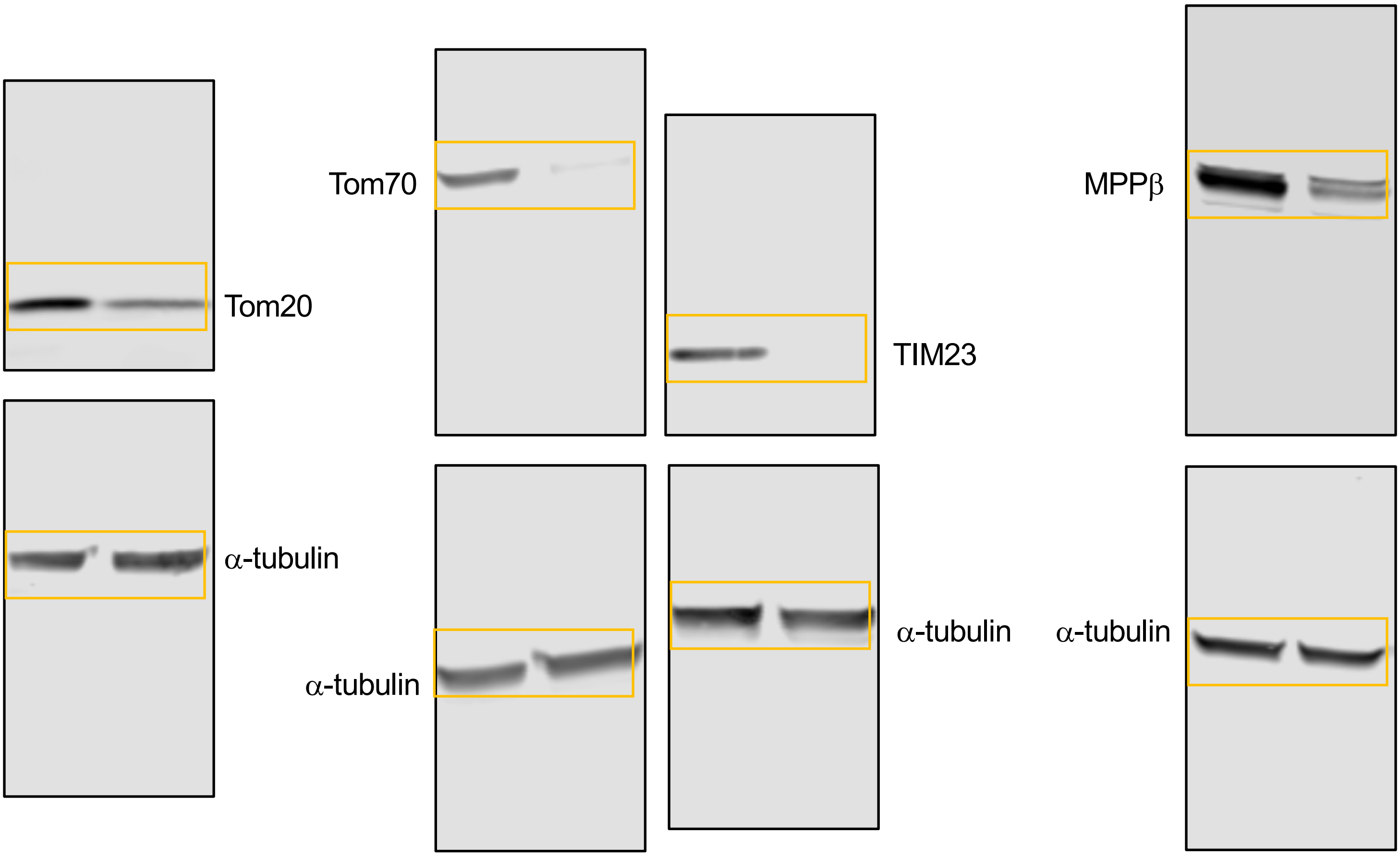

Figure S1E

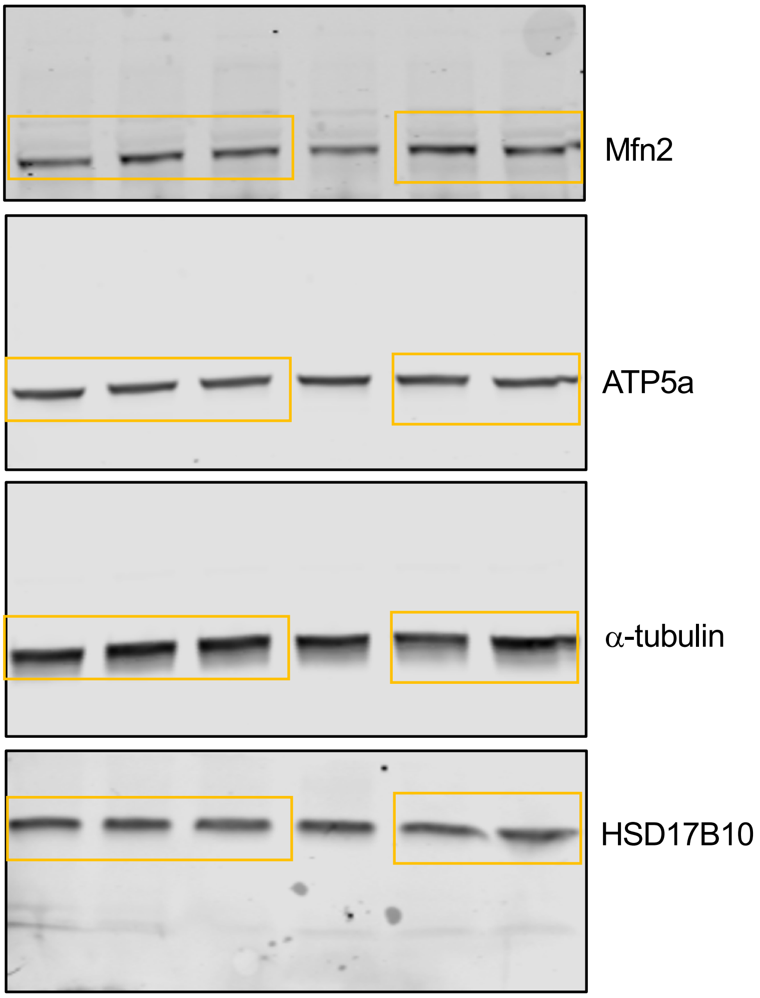

Figure S1F

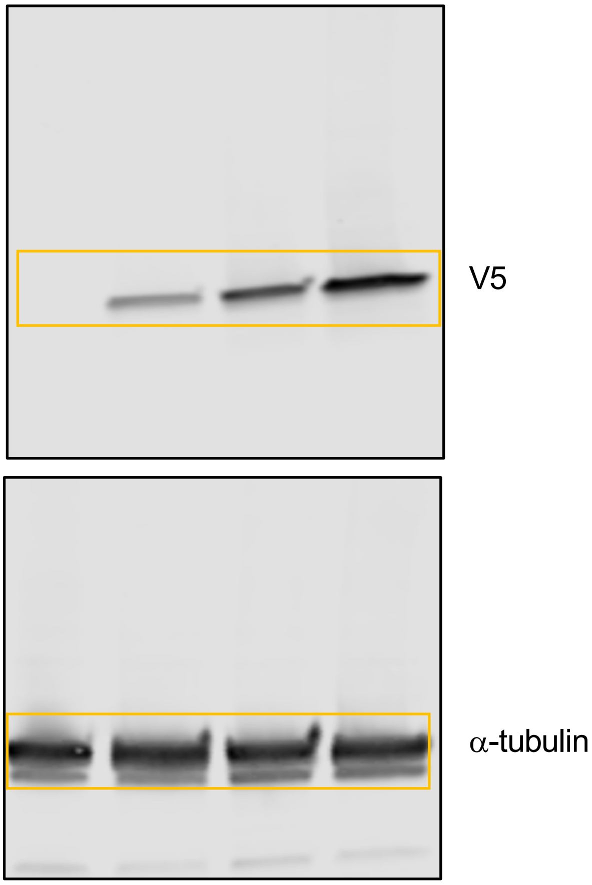

Figure S2D

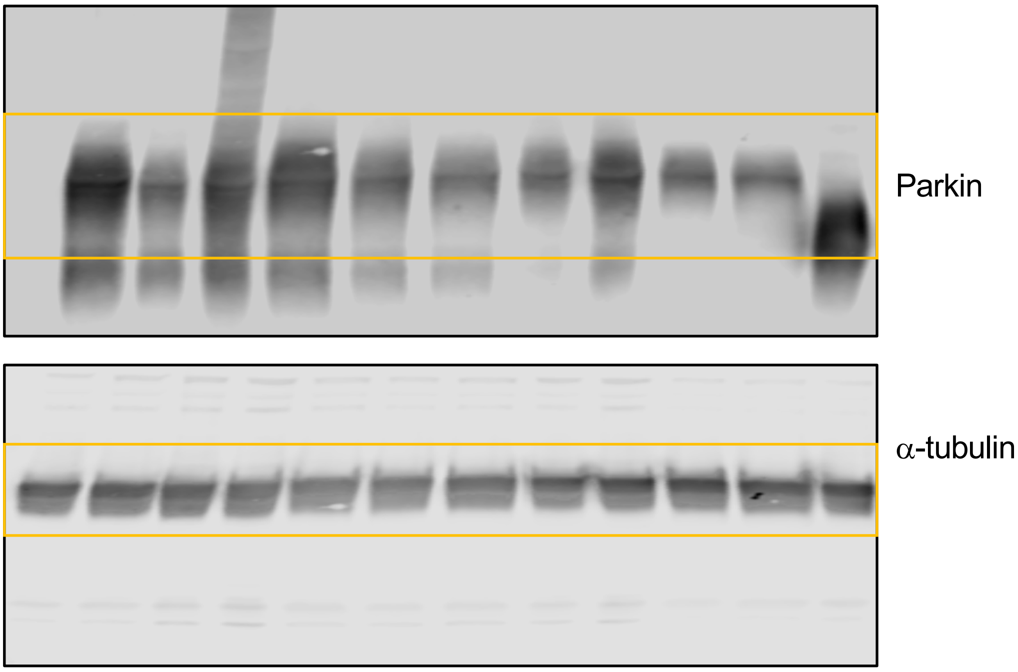

Figure S2E

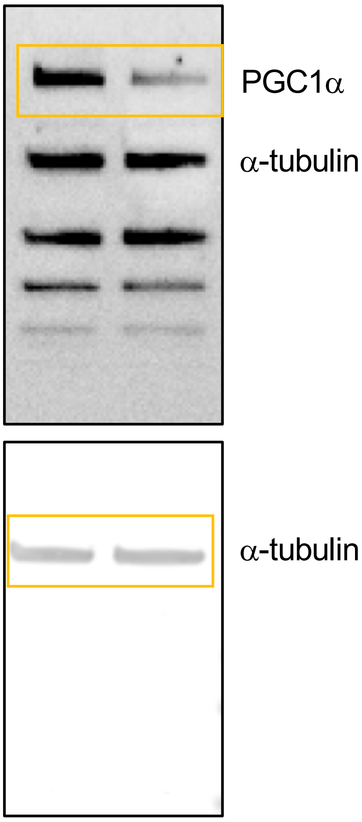

Figure S2F

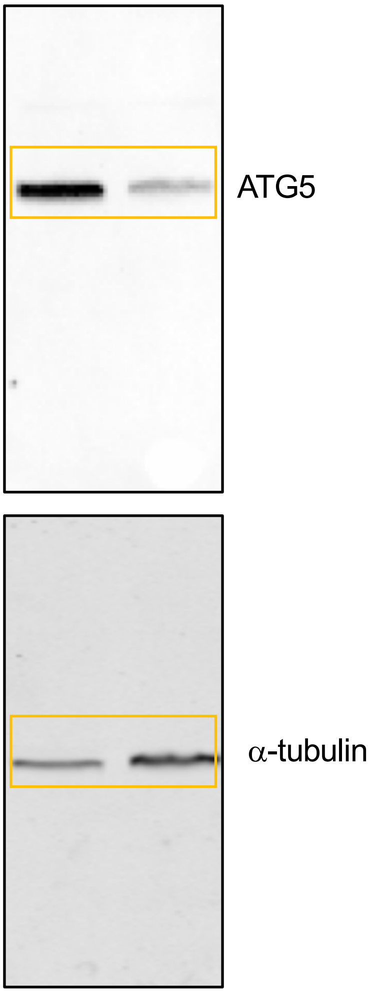

Figure S2G

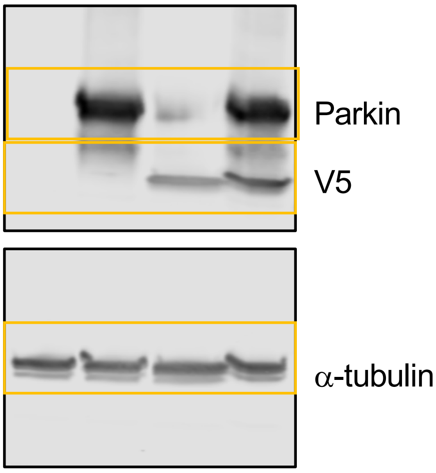

Figure S3A

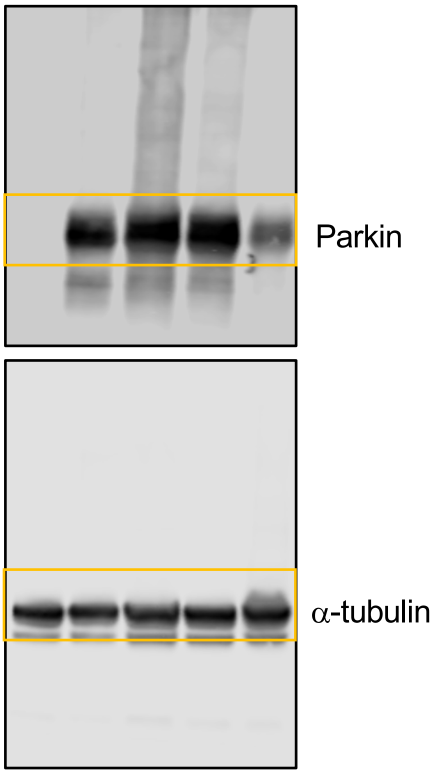

Figure S3B

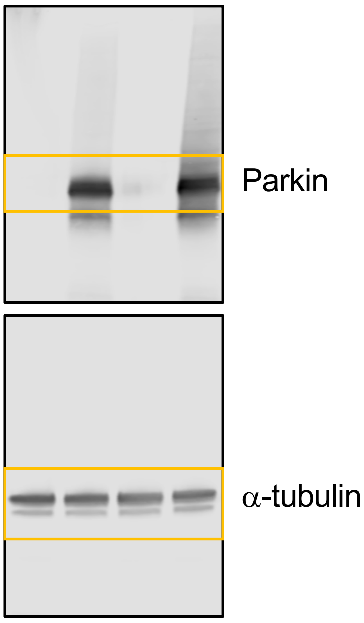

Figure S3C

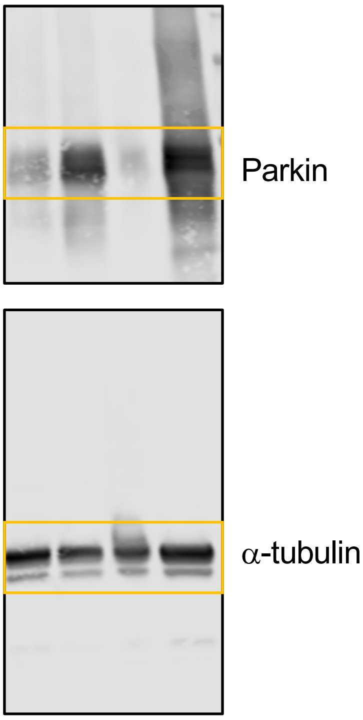

Figure S3D

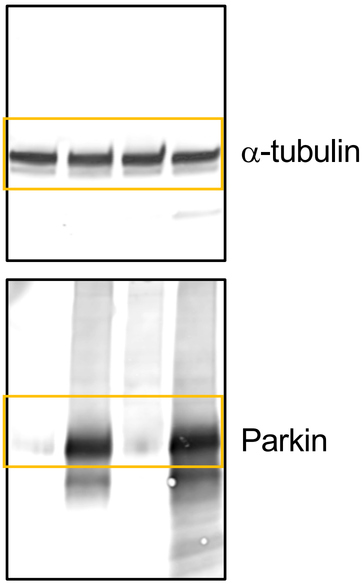

Figure S4B

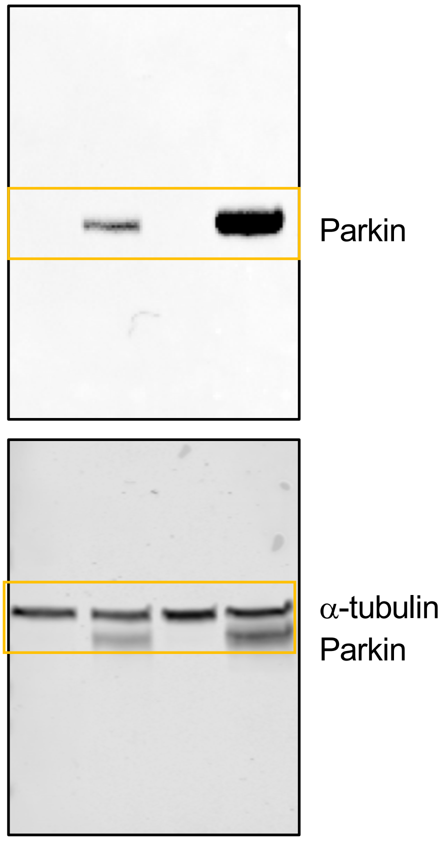

Figure S4E

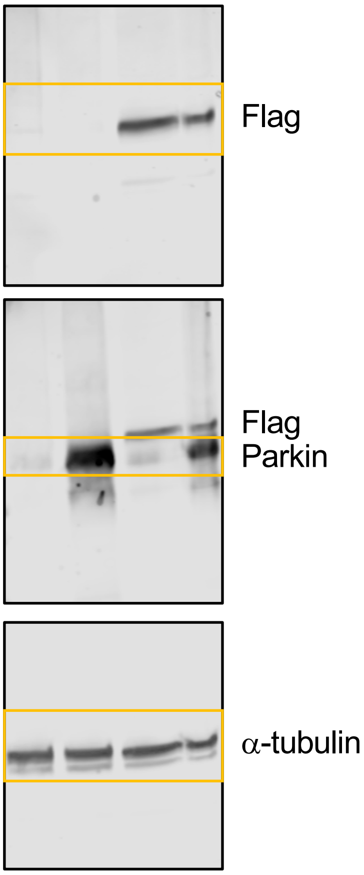

Figure S4F

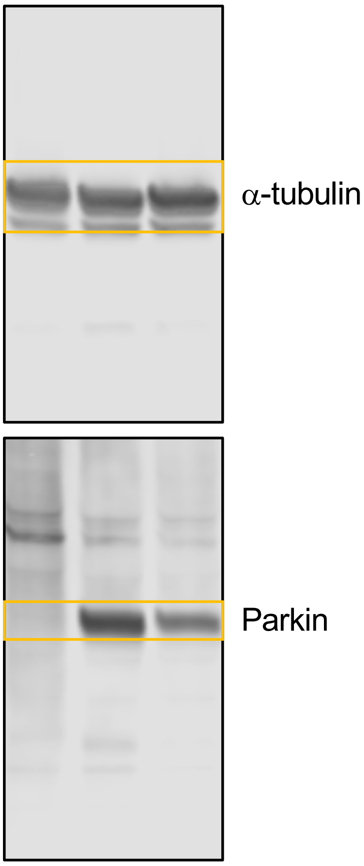

The PINK1 kinase-driven ubiquitin ligase Parkin promotes mitochondrial protein import through the presequence pathway in living cells

M. Jacoupy, E. Hamon-Keromen, A. Ordureau, Z. Erpapazoglou, F. Coge, J-C. Corvol, O. Nosjean, C. Mannoury La Cour, M. J. Millan, J. A. Boutin, J. W. Harper, A. Brice, D. Guedin, C. A. Gautier, O. Corti

Supplementary Tables:

Individual	Age	Sex	Gene	Mutation(s)	Protein
Patient 1	46	F	*PARK 2*	c.[(871+1_872-1)_(1083+1_1084-1)del];[125G>C]	p.[Ala291ValfsX8];[Arg42Pro]
Patient 2	40	F	*PARK 2*	c.[(871+1_872-1)_(1083+1_1084-1)del];[125G>C]	p.[Ala291ValfsX8];[Arg42Pro]
Patient 3	51	F	*PARK 2*	c.[(871+1_872-1)_(1083+1_1084-1)del];[125G>C]	p.[Ala291ValfsX8];[Arg42Pro]
Patient 4	50	M	*PARK 2*	c.[(534+1_535-1)_(618+1_619-1)del];[1321T>C]	p.[Gly179_Ala206del];[Cys441Arg]
Patient 5	45	F	*PARK 2*	c.[(412+1_413-1)_(534+1_535-1)del];[155delA]	p.[Ala138GlyfsX7];[Asn52MetfsX29]
Patient 6	37	F	*PARK 2*	c.[(171+1_172-1)_(734+1_735-1)del];[171+1_172-1)_534+1_535-1)del]	p.[Asn58GlufsX29];[Asn58_Gln178del]
Patient 7	65	M	*PARK 2*	c.[(7+1_8-1)_(171+1_7172-1)del];[101_102delAG]	p.[Val3GlufsX3];[Gln34ArgfsX5]
Patient 8	53	F	*PINK1*	c.[1366C>T];[1366C>T]	p.[Gln456*];[Gln456*]
Patient 9	52	F	*PINK1*	c.[1366C>T];[1366C>T]	p.[Gln456*];[Gln456*]
Control 1	53	M	-	No mutation	Normal
Control 2	32	F	-	No mutation	Normal
Control 3	54	M	-	No mutation	Normal
Control 4	65	F	-	No mutation	Normal
Control 5	64	F	-	No mutation	Normal
Control 6	52	M	-	No mutation	Normal

Supplementary Table 1. Characteristics of the donors of the primary skin fibroblasts used in the Study.

Primary antibodies			
**Type**	**Company**	**Catalog #**	**Host**
c-Myc	Santa Cruz	sc-40	Mouse
Flag	Sigma	F1804	Mouse
HSD17B10	abcam	ab10260	Mouse
Mfn2	abcam	ab50838	Rabbit
MPPβ	Proteintech	16064-1-AP	Rabbit
OPA1	BD Biosciences	612607	Mouse
Parkin	Millipore	mab5512	Mouse
PGC-1-α	Abcam	Ab54481	Rabbit
TIM23	BD Biosciences	611223	Mouse
TOM20	Abcam	ab56783	Mouse
TOM70	Abcam	ab106193	Mouse
Total OxPhos	Invitrogen	458099	Mouse
TuJ1	Covance	MMS-435P	Mouse
TuJ1	Covance	MRB-435P	Rabbit
Ubiquitin	Santa Cruz	sc-8017	Mouse
V5	Abcam	Ab27671	Mouse
α-tubulin	Abcam	ab7291	Mouse
α-tubulin	Abcam	ab18251	Rabbit
**Secondary antibodies**			
IRDye 800CW-conjugated anti-mouse/rabbit	Li-Cor	926-32211/926-32210	Goat
IRDye 680RD-conjugated anti-mouse/rabbit	Li-Cor	26-68071/926-68070	Goat
Anti-mouse/rabbit-HRP Fab fragment	GE Health Care	NA931-1ML/ NA9340-1ML	Sheep/Donkey
HRP-conjugated anti-mouse/rabbit	Jackson ImmunoResearch	115-035-146/111-035-144	Goat
Alexa 488-conjugated anti-mouse/rabbit IgG	Invitrogen	A32723/ A-11034	Goat

Supplementary Table 2. Antibodies used for immunofluorescence and western blotting.

Gene	RT-PCR forward primers	RT-PCR reverse primers
PARK2	5’-CGCAACAAATAGTCGGAAC-3’	5’-GAGGGTCGTGAACAAACTG-3’
PINK1	5’-GCAGCTACCAAGAGGCTCAG-3’	5’-CACCCCAGAGGCTTAGATGA-3’
ACTB	5’-GGACTTCGAGCAAGAGATGG-3’	5’-AGCACTGTGTTGGCGTACAG-3’

Supplementary Table 3. Sequence of primers used for quantitative RT-PCR analysis.

The PINK1 kinase-driven ubiquitin ligase Parkin promotes mitochondrial protein import through the presequence pathway in living cells

M. Jacoupy, E. Hamon-Keromen, A. Ordureau, Z. Erpapazoglou, F. Coge, J-C. Corvol, O. Nosjean, C. Mannoury La Cour, M. J. Millan, J. A. Boutin, J. W. Harper, A. Brice, D. Guedin, C. A. Gautier, O. Corti

Supplementary Methods

UB-AQUA/PRM Proteomics. UB-AQUA/PRM was performed essentially as described previously but with several modifications[1-3]. A collection of 21 heavy-labeled reference peptides[2,3], each containing a single $^{13}C/^{15}N$-labeled amino acid, was produced at Cell Signaling Technologies and quantified by amino acid analysis. UB-AQUA peptides from working stocks [in 5% (vol/vol) FA] were diluted into the digested sample [in 1% (vol/vol) FA] to be analyzed to an optimal final concentration predetermined for individual peptide. Samples and AQUA peptides were oxidized with 0.05% hydrogen peroxide for 20 min, subjected to C18 StageTip and resuspended in 1% (vol/vol) FA. MS data were collected sequentially by LC/MS on a Q Exactive mass spectrometer (Thermo Fisher Scientific) coupled with a Famos Autosampler (LC Packings) and an Accela600 LC pump (Thermo Fisher Scientific). Peptides were separated on a 100-µm i.d. microcapillary column packed with ~0.5 cm of Magic C4 resin (5 µm, 100 Å; Michrom Bioresources) followed by ~20 cm of Accucore C18 resin (2.6 µm, 150 Å; Thermo Fisher Scientific). Peptides were separated using a 60-min gradient of 3–25% ACN in 0.125% FA with a flow rate of ~300 $nL \cdot min^{-1}$. The scan sequence began with an Orbitrap full MS^1 spectrum with the following parameters: resolution of 70,000, scan range of 200–1,000 Thomson (Th), AGC target of $1x10^6$, maximum injection time of 250 ms, and profile spectrum data type. This scan was followed by 12 targeted MS^2 scans selected from a scheduled inclusion list with a 6-min retention time window. Each targeted MS^2 scan consisted of high-energy collision dissociation (HCD) with the following parameters: resolution of 17,500, AGC of $1x10^5$, maximum injection time of 200 ms, isolation window of 1 Th, normalized collision energy (NCE) of 23, and profile spectrum data type. Raw files were searched, and precursor and fragment ions were quantified using Skyline version 3.5[4]. Data generated from Skyline were exported into an Excel spreadsheet for further analysis as previously described[2]. Total UB was determined as the average of the total UB calculated for each individual locus, unless specified otherwise.

Mitochondrial import assay. In vitro translated proteins were incubated with mitochondria isolated from murine hearts as previously described[5], in the presence or absence of 2 µM CCCP at 30°C or 4°C for 1h in import buffer (80 mM KCl, 2 mM MgAc2, 10 mM Hepes pH

7.4 ATP-succinate). Following import, the reaction was layered on top of 500 µl of 250 mM sucrose, 10 mM Hepes pH 7.5 in a microfuge tube. The cushion was centrifuged (10,000 x g for 5 min at 4°C) and the mitochondrial pellet was digested with 50 µg/ml PK. PMSF (2 mM) was added to stop the reaction. Mitochondria were centrifuged (9,000 x g, 10 min) and subjected to SDS-PAGE together with inputs.

Supplementary references:

1 Phu, L. *et al*. Improved quantitative mass spectrometry methods for characterizing complex ubiquitin signals. *Mol Cell Proteomics* **10**, M110 003756, doi:10.1074/mcp.M110.003756 (2011).

2 Ordureau, A. *et al*. Quantitative proteomics reveal a feedforward mechanism for mitochondrial PARKIN translocation and ubiquitin chain synthesis. *Molecular cell* **56**, 360-375, doi:10.1016/j.molcel.2014.09.007 (2014).

3 Ordureau, A. *et al*. Defining roles of PARKIN and ubiquitin phosphorylation by PINK1 in mitochondrial quality control using a ubiquitin replacement strategy. *Proceedings of the National Academy of Sciences of the United States of America* **112**, 6637-6642, doi:10.1073/pnas.1506593112 (2015).

4 MacLean, B. *et al*. Skyline: an open source document editor for creating and analyzing targeted proteomics experiments. *Bioinformatics* **26**, 966-968, doi:10.1093/bioinformatics/btq054 (2010).

5 McBride, H. M., Millar, D. G., Li, J. M. & Shore, G. C. A signal-anchor sequence selective for the mitochondrial outer membrane. *The Journal of cell biology* **119**, 1451-1457 (1992).
